# Inter-locus as well as intra-locus heterogeneity in LINE-1 promoter methylation in common human cancers suggests selective demethylation pressure at specific CpGs

**DOI:** 10.1186/s13148-015-0051-y

**Published:** 2015-03-01

**Authors:** Nicole Nüsgen, Wolfgang Goering, Albertas Dauksa, Arijit Biswas, Muhammad Ahmer Jamil, Ioanna Dimitriou, Amit Sharma, Heike Singer, Rolf Fimmers, Holger Fröhlich, Johannes Oldenburg, Antanas Gulbinas, Wolfgang A Schulz, Osman El-Maarri

**Affiliations:** Institute of Experimental Hematology and Transfusion Medicine, University of Bonn, Sigmund-Freud Str. 25, 53127 Bonn, Germany; Department of Urology, Medical Faculty, Heinrich-Heine-University, Moorenstr. 5, 40225 Düsseldorf, Germany; Institute for Digestive Research, Lithuanian University of Health Sciences, Eiveniu g. 2, Kaunas, 50009 Lithuania; Bonn-Aachen International Center for IT (B-IT) Algorithmic Bioinformatics, University of Bonn, Dahlmannstr. 2, 53113 Bonn, Germany; Institute of Medical Biometry, Informatics and Epidemiology (IMBIE), University of Bonn, Sigmund-Freud-Straße 25, D-53127 Bonn, Germany

**Keywords:** LINE-1, L1 loci, DNA methylation, Methylation patterns, Cancer, Global methylation, Pyrosequencing, Bisulfite sequencing, Cancer classification

## Abstract

**Background:**

Hypomethylation of long interspersed element (LINE)-1 has been observed in tumorigenesis when using degenerate assays, which provide an average across all repeats. However, it is unknown whether individual LINE-1 loci or different CpGs within one specific LINE-1 promoter are equally affected by methylation changes. Conceivably, studying methylation changes at specific LINE-1 may be more informative than global assays for cancer diagnostics. Therefore, with the aim of mapping methylation at individual LINE-1 loci at single-CpG resolution and exploring the diagnostic potential of individual LINE-1 locus methylation, we analyzed methylation at 11 loci by pyrosequencing, next-generation bisulfite sequencing as well as global LINE-1 methylation in bladder, colon, pancreas, prostate, and stomach cancers compared to paired normal tissues and in blood samples from some of the patients compared to healthy donors.

**Results:**

Most (72/80) tumor samples harbored significant methylation changes at at least one locus. Notably, our data revealed not only the expected hypomethylation but also hypermethylation at some loci. Specific CpGs within the LINE-1 consensus sequence appeared preferentially hypomethylated suggesting that these could act as seeds for hypomethylation. *In silico* analysis revealed that these CpG sites more likely faced the histones in the nucleosome. Multivariate logistic regression analysis did not reveal a significant clinical advantage of locus-specific methylation markers over global methylation markers in distinguishing tumors from normal tissues.

**Conclusions:**

Methylation changes at individual LINE-1 loci are heterogeneous, whereas specific CpGs within the consensus sequence appear to be more prone to hypomethylation. With a broader selection of loci, locus-specific LINE-1 methylation could become a tool for tumor detection.

**Electronic supplementary material:**

The online version of this article (doi:10.1186/s13148-015-0051-y) contains supplementary material, which is available to authorized users.

## Background

Tumors are distinguished by extensive epigenetic deregulation, of which changes in DNA methylation are best characterized. In many tumor tissues, widespread ‘global’ hypomethylation and locus-specific or regional hypermethylation has been simultaneously observed. It is generally believed that decreased methylation at repetitive elements, such as short interspersed elements (SINEs) and long interspersed elements (LINEs), which collectively constitute about 33% of the human genome [1] significantly contributes to global hypomethylation in cancer cells. DNA methylation changes at LINE-1 elements, in particular, have been reported for almost all tumor types [2-11]. Notably, while LINE-1 hypomethylation has been detected in many common cancers, its extent and its relationship to clinical parameters is quite diverse [12,13]. In addition to changes in cancer tissues, LINE-1 methylation in blood and other body fluids has also been suggested as a biomarker for monitoring cancer risk [14].

Overall, LINE-1 repeats constitute about 17% of the human genome [1]. Although most repeats are fragmented, a significant fraction retains full length (>6 KB). Among these full-length elements, an estimated 800 copies per haploid genome of human-specific LINE-1 (L1Hs subtype) retain a relatively intact promoter with comparatively high CpG-density. In agreement with other authors, we have demonstrated that in healthy total blood, methylation levels are not uniform across all loci and that LINE-1 repeats are less methylated on the inactive X chromosome than on the active X [15] leading to higher overall LINE-1 methylation levels in males [16,17]. However, no detailed data on the variability of methylation of individual LINE-1 elements in disease are available, especially not in common cancers.

Consequently, it is an open question whether any methylation changes at L1Hs promoter regions might provide reliable biomarkers for detection, characterization, and classifications of different cancers. Moreover, it is unknown whether individual LINE-1 loci and different CpG sites within one locus are affected by methylation changes to the same degree and whether changes in LINE-1 methylation occur in a random or coordinate manner. Importantly, methylation changes at specific LINE-1 loci may provide more information than global LINE-1 methylation for diagnostic purposes, while specific loci could behave differently depending on the tumor type. Finally, it is thought that LINE-1 hypomethylation may lead to increased transcriptional activity [12,13,18]. Therefore, in this study, we analyzed 11 (five autosomal and six X-linked) specific LINE-1 loci in five different common human cancers and compared their methylation to the results for overall LINE-1 methylation from global degenerate assays. The specific loci were chosen based on their high homology to the L1Hs reference sequence (L1Hs; National Center for Biotechnology Information (NCBI) accession number: X58075) as previously described [15]. Our aim was - on the one hand - to ascertain the detailed methylation changes of individual CpGs at these specific LINE-1 loci in tumor tissues and patient blood and - on the other hand - to explore whether methylation changes at specific loci might be more suitable for diagnostic purposes than overall changes measured by degenerate assays.

Our results highlight the variability of methylation deregulation at individual LINE-1. Interestingly, in addition to hypomethylation, hypermethylation was also observed at some elements in tumor tissues but rarely in blood cells from tumor patients. Moreover, next-generation sequencing revealed preferential hypomethylation at focal CpG sites, suggesting that these may act as ‘seeds’ for hypomethylation. *In silico* modeling showed that at such (hypomethylated) CpG sites, C5 of the cytosine ring, which is subject to methylation, tends to be present in the major DNA groove facing toward the nucleosome proteins. No individual LINE-1 locus reliably distinguished all tumors from normal tissues or blood from healthy persons vs. tumor patients. However, most tumor samples showed significant hypomethylation or hypermethylation of at least one LINE-1 locus. Thus, studying methylation at LINE-1 loci could become a useful tool to identify tumor samples. However, more informative single LINE-1 loci should be identified in larger studies before designing cost-effective clinical tests based on locus-specific LINE-1 methylation.

## Results

We analyzed DNA methylation in the CpG-rich promoters of 11 specific LINE-1 (L1Hs) elements from five autosomal and six X-linked loci (Additional file 1) by bisulfite pyrosequencing. These loci were chosen due to their high homology to the L1Hs consensus sequence (L1Hs; NCBI accession number: X58075) [15]. In addition, a previously described degenerate assay [16] was used to investigate two CpG sites in the L1Hs consensus sequence in a genome-wide fashion.

### LINE-1 locus-specific methylation is moderately tissue-specific in normal tissues

**Figure 1:**
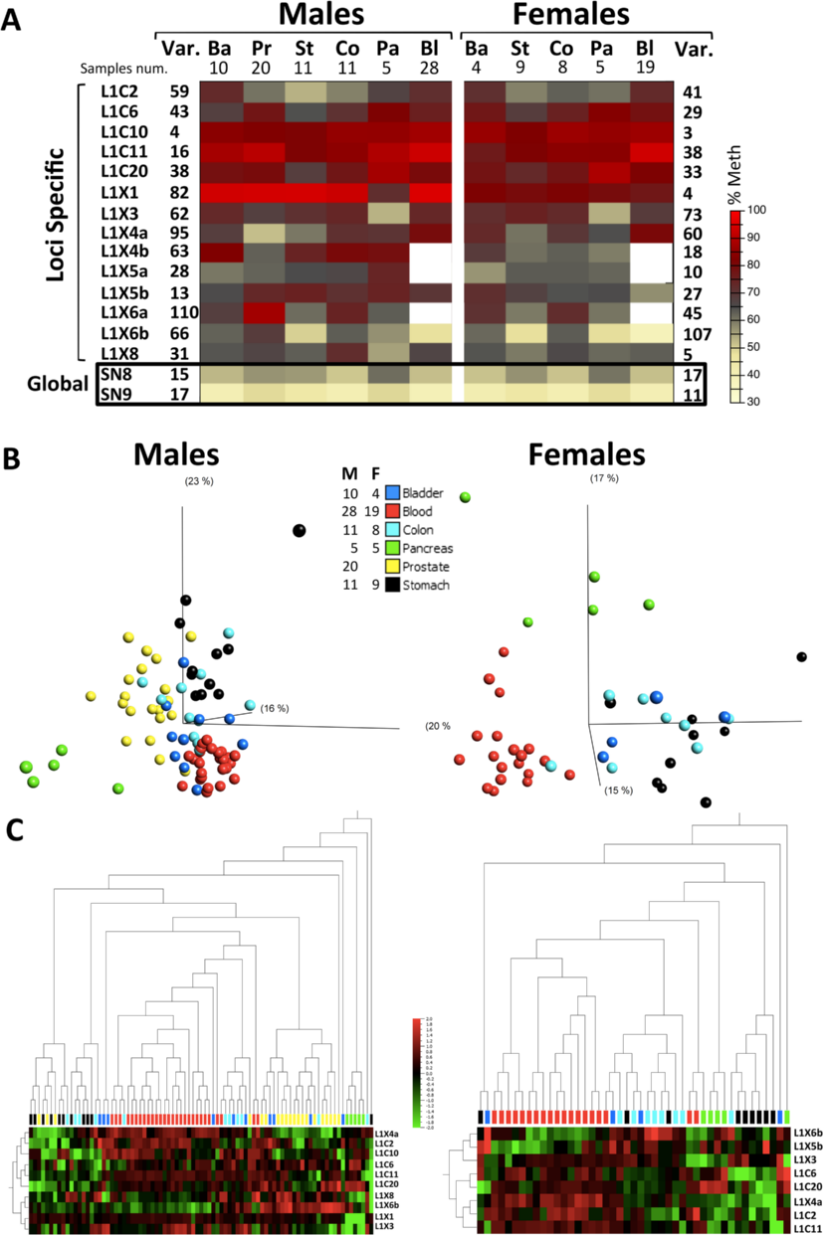
**Tissue-specific methylation at specific LINE-1 loci (from morphologically normal tissue). (A)** Heat map of methylation means at different LINE-1 loci in all tissues and in blood as measured by bisulfite pyrosequencing. The variances of mean methylation for males and females, across the studied tissues, are shown on the left and right of the graph, respectively. Global LINE-1 methylation values are given at the last two rows of the heat map (in black frame). The numbers of samples are shown under the tissue denomination. **(B)** Principle component analysis of methylation at all specific LINE-1 loci demonstrating identification of several separate tissues entities (ANOVA *p* = 0.0107 for males and *p* = 0.0031 for females; at a false discovery rate of 5%). The numbers of samples are shown to the left. **(C)** Hierarchical clustering of the individual methylation values for all samples. Ba: bladder; Bl: blood; Co: colon; F: female; M: male, Pa: pancreas; Pr: prostate; St: stomach; Var.: variance.

Tissue-specific variation in methylation was obvious from comparisons of mean values (Figure 1A); a detailed locus-by-locus ANOVA yielded positive statistical results at most loci in male and female samples, indicating general tissue-specific methylation of LINE-1 elements (Additional file 2).

Principle component analysis (PCA) used at a false discovery rate of 5% visualizes the separation between different normal tissues by the methylation levels of specific LINE-1 loci (Figure 1B). In male samples, the blood, prostate, and pancreas could be distinguished as specific entities (ANOVA *p* = 0.0107), while in female samples, the blood and pancreas samples also formed relatively separate entities (ANOVA *p* = 0.0031). The samples from the colon, stomach, and bladder appeared more intermixed with no clear entities forming. Unsupervised hierarchical clustering (Figure 1C) essentially replicated the PCA results, further highlighting that the L1X6b locus is hypomethylated in the blood, the L1C20 locus is hypermethylated in the pancreas, and the L1X4a locus is hypomethylated in the prostate relative to other tissues.

In the global LINE-1 methylation assays (Figure 1A; Additional file 2), inter-tissue variability was also evident but the mean differences were smaller compared to those at individual loci. Nevertheless, in male samples, seven (for SN8) and six (for SN9) significant differences between pairs of different tissues were detected, i.e., more differences than for any single specific locus.

Since the analyzed healthy tissues were obtained from tumor patients and were adjacent to tumor tissues, their methylation levels might, conceivably, also be abnormal. Therefore, we compared the methylation at three loci each in the colon (L1C2, L1C10, and L1C11) and in the stomach (L1C10, L1C20, and L1X3) for histologically normal tissues from tumor patients and normal control tissues obtained from non-tumor individuals undergoing surgery because of obesity (for stomach samples) or hemorrhoids/prolapsus recti (for colon samples). No statistically significant differences were observed between the two groups of samples (Additional file 3). Thus, methylation of individual LINE-1 loci appears to be broadly unchanged in morphologically normal tissues adjacent to cancers.

### Most LINE-1 loci undergo changes in methylation in tumor tissues

**Figure 2:**
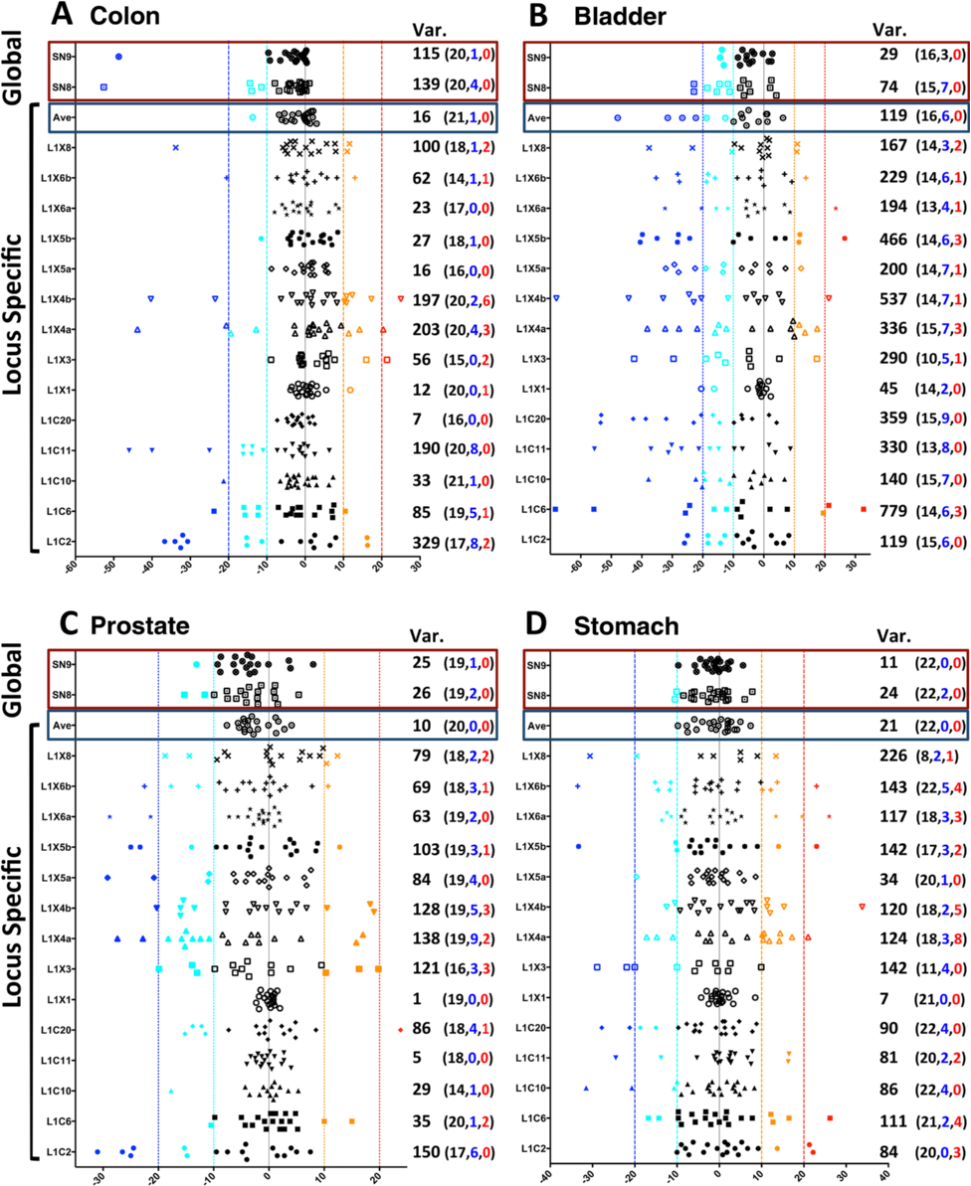
**Methylation differences between paired tumor tissues and neighboring morphologically normal tissues.** Differences in methylation as measured by bisulfite pyrosequencing are plotted for all paired samples from **(A)** colon, **(B)** bladder, **(C)** prostate, and **(D)** stomach. The red and black frames at the top of each panel enclose the results from the global methylation assay and the average methylation of all individual LINE-1 loci, respectively. Methylation differences between < −20, −20 to −10, −10 to 10, 10 to 20 and >20 are marked as blue, turquoise, black, orange, and red symbols, respectively. The variance of methylation differences is shown to the right of each locus. Values in parenthesis correspond to the total number of samples (black, left), number of samples below the −10 threshold (blue, center) followed by number of samples above the +10 threshold (red, right). Var.: variance.

In the following sections, we conservatively define hypomethylation and hypermethylation as a more than 10% increase and a 10% decrease in absolute methylation, respectively. When plotting the methylation differences between paired samples of tumor and healthy tissues (Figure 2; Additional file 4), the following points are noticed: 1) hypomethylation as well as hypermethylation at individual LINE-1 loci is observed in tumor samples and hypo- and hypermethylation were not mutually exclusive for some LINE-1 loci, 2) almost all LINE-1 loci have methylation changes in at least one sample, 3) locus-specific values frequently exceed 10% absolute change in methylation but rarely overall LINE-1 methylation as measured by the global assays.

### Most tumor samples contain at least one hyper- or hypomethylated locus

Of all 80 paired cancer samples, 72 (90%) harbored methylation changes on at least one locus. Thirty-four cancer tissues had only hypomethylated loci and 14 tumor samples had only hypermethylated loci, whereas 24 samples contained both hypomethylated and hypermethylated loci (Additional file 5A). The number of loci-affected samples ranged from 0 (in eight cancer tissues) to 12 (Additional file 5B). Generally, the highest number of simultaneously affected loci was seen in bladder cancer samples.

In order to determine the best LINE-1 locus to distinguish tumor from non-tumor samples by methylation, we applied a multivariate logistic regression model on both, the locus-specific data (14 studied loci) and the global data (SN8 and SN9 global assay). Markers with statistically significant difference between tumor and healthy tissues (Wilcoxon signed rank test, *p* values corrected for multiple testing using Benjamini and Hochberg’s method) were included in this model. As determined by the area under curve (AUC) (Additional file 6), locus-specific data and the global methylation values could distinguish tumor from normal samples comparably well.

### Correlation between methylation levels and clinical characteristics

We further analyzed systematically the correlations of the available clinico-pathological parameters for each tumor type with the methylation of the individual LINE-1 elements as well as the total LINE-1 methylation (SN8 and SN9) (Additional file 7). No widespread significant correlation between LINE-1 methylation and the clinical parameters was evident. In particular, the correlations with statistically significant values (*ρ* > 0.7 or *ρ* < −0.7 with *p* < 0.01 for Spearman correlation or *p* < 0.01 for Mann-Whitney test or Krustal-Wallis test) numbered 0 for bladder, 4 for colon, 6 for pancreas, 1 for prostate, and 1 for stomach cancers. After considering Bonferroni adjustment for multiple testing (which requires *p* < 0.00294 for significance), only two correlations remain significant, namely methylation at L1X6a with the number of removed lymph nodes in female colon cancers and L1X6b methylation with age in female pancreatic cancer. A correlation between methylation levels and age was observed in tumor samples at several loci but did not remain significant after adjustment for multiple testing.

### Assessment of locus-specific LINE-1 expression in prostate cancer

For eight of the 11 single LINE-1 loci assessed for methylation, specific PCR assays for expression analyses could be designed. Expression of these individual LINE-1 elements was first tested in a panel of prostate and bladder cancer cell lines using standard end point RT-PCR. In prostate cancer cell lines, five LINE-1 elements showed no detectable expression, while transcripts from three LINE-1 loci (L1C2, L1C11, and L1X4) were detectable. In contrast, no expression of any specific LINE-1 element was detectable in a large panel of bladder cancer cell lines (data not shown) despite frequent and generally increased overall LINE-1 expression in these cells [19]. Subsequently, expression of these LINE-1 elements was assessed in a set of 12 benign and 47 prostate tumor tissues by quantitative reverse transcription (qRT)-PCR [20]. While expression of L1C2 was weak in benign tissues, it increased slightly but significantly in prostate cancer tissues (Additional file 8; Mann-Whitney *U*-test; *p* = 0.023). Likewise, expression of L1C11 was significantly elevated in tumor tissues (Additional file 6; Mann-Whitney *U*-test; *p* < 0.001), whereas expression in all normal tissues was near the limit of detection. Expression of L1X4 tended to be higher in tumor tissues without reaching statistical significance (Additional file 6; Mann-Whitney *U*-test; *p* = 0.145).

Interestingly, two of the three LINE-1 loci expressed in prostate cancer, i.e., L1C2 and L1X4a, are less strongly methylated than others, even in normal prostate tissues (Additional file 2), and undergo additional significant decreases in DNA methylation in prostate cancer (Additional file 4D).

### Comparison of locus-specific methylation in blood cells between patients and healthy controls

Peripheral blood samples were available from pancreatic, gastric, and colon cancers. Blood samples were obtained on the day of the surgery before the start of the treatment. This allowed comparison to blood samples from healthy individuals without known history of cancer, matched by age and gender. A statistically significant difference in methylation between cancer cases and controls was obtained in male patients with pancreatic carcinoma at L1C2, L1C20, L1X4a, L1X5b, and L1X8; while in female patients, only L1X4a was significant (Additional file 9). Regarding stomach cancer, only L1X8 in male patients and L1C6 in female patients were significantly different. Finally, in colon cancer cases, only L1X5b methylation was significantly different in female patients compared to healthy women. Thus, predominantly male patients with pancreatic cancer had significant methylation differences at several LINE-1 loci in blood cells distinguishing them from healthy blood donors.

In accordance with the above results, linear regression analysis identified the best methylation markers to distinguish between blood samples from tumor patients and healthy persons as L1X8 for colon and L1X8 as well as L1X4A for pancreatic and stomach cancers. When comparing the utility of these locus-specific markers to global LINE-1 methylation by receiver operating characteristic (ROC) analysis, the AUC was markedly larger for the specific markers in comparison to the global assay (pancreas, specific: 0.85, global: 0.62; stomach, specific: 0.83, global: 0.54) (Additional file 10).

### Confirmation and depiction of the nature of methylation changes by massive parallel sequencing

Having identified widespread changes in methylation levels at specific LINE-1 loci in tumors, we wanted to find answers to two obvious questions regarding the nature and patterns of these changes: 1) Are the hypomethylation or hypermethylation changes allelic or mosaic in nature? 2) Do the methylation changes affect all CpGs within one individual LINE-1 promoter region to the same degree or are there intra-regional differences, whereby some CpGs are more prone to methylation changes?

To this end, we performed massive parallel sequencing for one pair of tumor-healthy samples from the prostate, bladder, stomach, and colon, choosing male samples that presented the highest numbers of hypo- or hypermethylated loci according to pyrosequencing.

**Figure 3:**
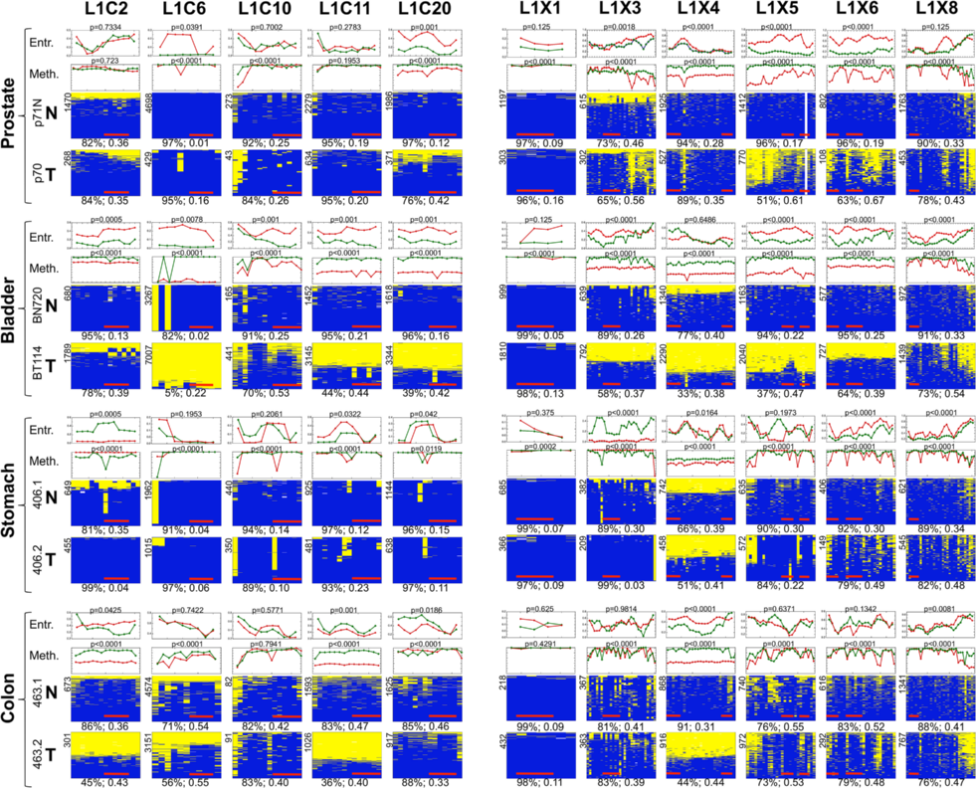
**Analysis of LINE-1 methylation by massive parallel sequencing.** Summary heat maps of the massive parallel sequencing analysis of the methylation of five specific autosomal (L1C2, L1C6, L1C10, L1C11, L1C20) and six specific X-linked (L1X1, L1X3, L1X4, L1X5, L1X6, L1X8) LINE-1 loci of four selected paired tumor and neighboring non-tumor tissue samples. Each heat map corresponds to the methylation patterns of one locus from one sample. Every line in the heat map corresponds to a sequence originating from an individual sequence read; each column represents a CpG site at the promoter of the specific LINE-1. Methylated CpGs are indicated in blue, unmethylated CpGs are marked in yellow. Below each heat map average methylation across all individual sequences and all CpG sites is given followed by the average methylation entropy. The number of sequences from which the heat map was constructed is indicated at the upper left side of the heat map. Horizontal red bars in the heat maps represent the regions studied by pyrosequencing. The graphs above each heat map represent the methylation average at every CpG site of the tumor tissue (red) and the morphologically normal tissue (green); *p* values for differences of methylation were calculated by Wilcoxon test. The diagrams at the top show the entropy (tumor in red, normal in green) in sliding windows of four CpGs with the corresponding *p* values (Wilcoxon test). Entr.: entropy; Meth.: methylation.

As predicted by pyrosequencing, most changes presented as hypomethylation of the respective tumor tissue (Figure 3). Three different patterns of methylation could be discerned by deep sequencing: 1) sequences that were uniformly methylated (such as L1C6 in normal prostate) or uniformly unmethylated (such as L1C6 in the bladder tumor); 2) allelic methylation, whereby the readings clearly fall into two groups, either methylated at (almost) all CpGs or unmethylated at all CpG, seen, e.g., at L1C2 and L1C11 in colon tumor samples; 3) alternating patches of methylation and demethylation that often appear consistent in most DNA molecules at a given locus thereby generating patterns with valleys and mountains; examples include L1C10 in bladder and colon tumors; L1X3 in prostate tumor and colon; L1X5 in prostate and colon tumors; and L1X6 in prostate, colon, and stomach tumors. The methylation entropy allows differentiation of the patchy methylation patterns by their increase and fluctuation in entropy. These entropy fluctuations are well illustrated by L1X5, L1X6, and L1X8 in the prostate cancer sample (Figure 3).

We also sequenced the same regions from male blood samples (one patient with pancreatic and gastric cancer and two patients with colon cancer, plus one pool from three healthy individuals). Several significant differences between the patient blood samples and the healthy blood samples were observed (Additional file 11). Prominent hypomethylation was observed at several loci and in most blood samples from tumor patients, with the most impressive change at locus L1X3 for the blood sample from a stomach cancer patient showing a reduction of nearly 50%. Hypermethylation was also observed but did not exceed 5%.

**Figure 4:**
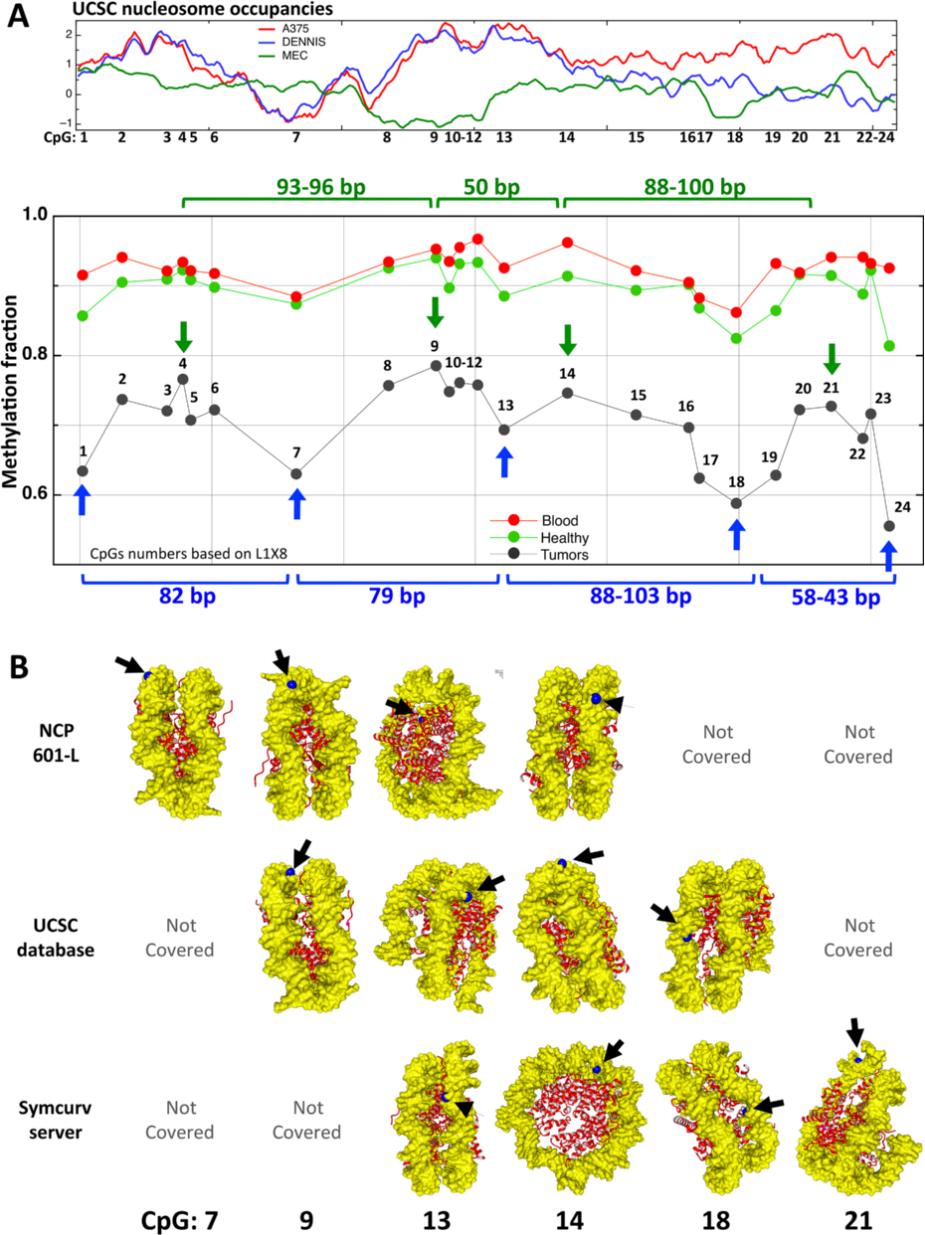
**Summary presentations of the relative location of hypomethylated CpG sites at five X-linked LINE-1 loci. (A)** The upper panel represents the predicted averaged nucleosomal occupancy of all five X-linked LINE-1s, based on the UCSC track: regulation, nucleosome occupancy [42]. The high peaks of red and blue lines (experimental results designated as A375 and DENNIS at the UCSC, respectively) correspond to a high probability of nucleosome occupancy, while the green peaks (designated as MEC) correspond to a low probability of nucleosome occupancy. In the lower panel, graphs represents the average methylation of all five X-linked LINE-1 loci for the six sequenced tumor samples (black), for their normal tissue controls (green), and for four blood controls (red). Vertical blue and green arrows represent the relatively hypo- and hypermethylated peaks (CpG numbering is according to L1X8). **(B)** The predicted localization of a particular cytosine carbon based on the three models of LINE-1 DNA around the histone octamers (as described in methods). The cytosine carbons in question are labeled in blue, histones are in red, and DNA is in yellow. The number of the CpGs whose **C** is labeled is shown at the lower part of the graph. NCP: nucleosome core particle; UCSC: University of California, Santa Cruz.

The detailed sequencing data highlight that at many LINE-1 loci not all CpG sites become equally strongly hypomethylated. Rather, some sites appear to be more susceptible to tumor-associated changes than others. For instance, the last CpG sites in L1X8 are clearly hypomethylated but not the preceding sequence of the element (Figure 3). By aligning all studied LINE-1 sequences from the six different samples analyzed by National Geodetic Survey (NGS) and calculating the average methylation at each CpG site per locus (Additional files 12 and 13), a substantial overlap becomes obvious among the CpGs that are hypomethylated across different samples, loci, and tissues. A similar overlap is seen among those CpGs that appear to be protected from demethylation. This observation is illustrated in Figure 4A for the X-linked loci, indicating a degree of periodicity as hypomethylated CpGs appear to be frequently separated by approximately 80 to 100 or 40 to 60 nucleotides (Figure 4A).

Such hypomethylated hotspots - if regular across different samples and different tissues - will create a defined pattern of methylation. When such patterns exist, a good degree of correlation (Spearman correlation) in methylation levels should be observed when all CpG sites of a given locus are simultaneously compared between different samples. Indeed, at some loci, inter-sample correlation was particularly strong. The long sequenced regions that contained larger number of simultaneously studied CpGs, such as L1X2, 3, 4, 5, and 8 (25 to 31 CpGs), showed stronger inter-sample correlations than the loci with lower numbers of CpGs, such as L1X1 (7 CpGs) and L1C2, 6, 10, 11, and 20 (12 to 15 CpGs) (Additional file 14).

### Non-repeat sequences also show unique methylation patterns

As these regularities in the methylation patterns at LINE-1 repeats have not been previously described, it has also not been investigated whether such patterns exist for single-copy sequences like CpG island gene promoters. We therefore took advantage of previously published data [21] from colon and breast cancers on the methylation of promoter regions of 77 single-copy genes to search for the existence and periodicity of such patterns (Additional file 15). We selected 15 regions based on the existence of intra-locus variability and determined the Spearman correlation values for inter-sample correlation of methylation values. Significant correlations would indicate the presence of a pattern across samples and tissues. Indeed, five regions showed obvious high levels of correlation across different samples as well as different (tumor as well as control) tissues (Additional file 15). Therefore, patterns of periodic hypo- and hypermethylation may not be unique for the LINE-1 repeats.

### Positional orientation of preferentially hypermethylated and hypomethylated CpGs on modeled nucleosomal structures

Since we found specific segments (or CpGs) hypomethylated across different loci, across several samples and across different tissues and since these particular CpGs appear to be non-randomly and to some degree regularly spaced within the LINE-1 sequence (Figure 4), we investigated whether these CpGs are positioned at specific sites in the nucleosome complex using three different *in silico* models. In each model, we could cover several CpG dinucleotides simultaneously, in particular, one hypomethylated (CpG 13) and one hypermethylated (CpG 14) CpG (Figure 4B). In general, we observed that cytosine C5 of a hypomethylated CpG is oriented towards the histones, while it is oriented away from the histones at a hypermethylated CpG.

### Inter-loci and intra-locus correlations

One important question is whether methylation changes occur at different LINE-1 loci in a random manner or in a more coordinated fashion. Inter-locus correlations were therefore calculated separately for the tumor samples and the corresponding healthy neighboring tissues (Additional file 16). In general, few inter-locus correlations were consistent in analyses of both tissue sets, indicating randomness in the degree of hypo- and hypermethylation at every individual locus. After applying the conservative Bonferroni correction for multiple testing, even fewer correlations remained significant. Stronger and more frequent positive correlations were present only in bladder tumor samples.

## Discussion

Since its discovery in the 1980s, a large number of studies have addressed DNA hypomethylation in human cancer. Although significant hypomethylation of repetitive sequences like LINE-1 is well established in many different cancers, no clinical use of overall or locus-specific LINE-1 methylation levels as diagnostic or prognostic biomarker has yet ensued. To date, most studies have used global assays that average methylation values of literally thousands of LINE-1 sequences dispersed throughout the genome to a single value. The major obstacle to clinical application derives from the considerable overlaps (and lack of accurate discrimination) in the total LINE-1 methylation levels between healthy samples and tumor samples in most studies [22], which limits the sensitivity and specificity of global LINE-1 hypomethylation assays. This motivated us to analyze methylation at 11 individual specific LINE-1 loci and to investigate the clinical usefulness of using single-loci-specific markers to discriminate between healthy and cancer conditions. In addition, massive parallel sequencing was applied to individual loci to reveal the exact architecture of the methylation changes.

### Characteristics of LINE-1 methylation change in different tumors

At present, only a small number of studies have reported on the methylation levels of individual LINE-1 repeats [23,24]. The scarcity of high-resolution locus-specific data is partially due to difficulties in specifically targeting assays to individual elements due to their highly similar and repetitive nature. One study used mostly cell lines and the assays covered only few CpG sites per repeat [23]. In another study, the bisulfite sequencing coverage seemed too low to deliver accurate quantitative data on individual repeats [24]. We therefore aimed to obtain highly accurate methylation measurements at single CpG resolution in different primary tumor samples, taking advantage of previously established amplification assays for specific L1Hs loci [15].

As expected, hypomethylation of different loci was the predominant observation, but pyrosequencing assays revealed considerable hypermethylation at several individual loci (Figure 2). Hypermethylation was restricted neither to a given locus nor to a specific tissue and was not mutually exclusive with the hypomethylation of other loci (Additional file 5). The massive parallel sequencing data showed that hypomethylation can occur as uniform allelic demethylation (where all CpGs in a given DNA molecule are hypomethylated) or can be patchy in appearance (where only some CpGs are hypomethylated) (Figure 3). These two types of methylation patterns are reflected in the methylation averages and in entropy, whereby allelic patterns of hypomethylation yield stable levels of both methylation and entropy, whereas patchy methylation patterns are reflected in unsteady entropy levels (compare Figure 3).

Strikingly, patchy hypomethylation often affects CpG sites in a non-random manner across patients and tissues (Figure 4, Additional file 13). Such non-randomly defined patterns suggest that some CpG sites more prone to hypomethylation act as ‘seeds’ initializing hypomethylation, whereas others remain protected from hypomethylation. These two types of CpGs could be defined by specific selective interactions with DNA binding proteins or by fixed nucleosome occupancy. A certain periodicity in the location of these sites hints at the latter possibility (see Figure 4). The observed periodicity is reminiscent of that of DNMT3A action on DNA [25]. However, DNMT periodicity is unlikely to contribute to this phenomenon at LINE-1 sequences, mainly because the observed periodicity at the LINE-1 is in the range of 40 to 100 bases (without fixed preference), whereas the periodicity in DNMT3a patterns of methylation is at a fixed scale of 8 to 10 bases.

*In silico* modeling of LINE-1 DNA in nucleosomes by three models suggests a different orientation bias between CpGs becoming hypo- or hypermethylated. In general, CpGs having the methylatable carbon of their cytosine base located in the major groove are oriented towards the histones and would be less accessible to methylation. This orientation, therefore, might - over time - favor hypomethylation. The converse reasoning applies to CpGs whose cytosine C5 faces away from the histones. In recent years, experimental data supporting this in/out bias for CpG methylation has accumulated. Genome-wide studies show that the minor grooves of unmethylated CpGs point away from the histones, thus cytosine C6 (subject to DNMT nucleophilic attack) located in the major groove, would be facing the histones [26-28]. Similarly, a recent study using nucleosome reconstitution experiments in conjunction with high throughput sequencing suggested that the minor grooves of most methylated CpGs are oriented towards the histone octamer, whereas the methylated fifth carbon position would be oriented away from the histones octamer [29]. DNA methyltransferases preferentially target the major groove of CpG dinucleotides, while methyl-binding proteins only interact with methylated CpGs in the DNA major groove [30-32]. This orientation bias could also underlie the tendency of particular CpG sites within the LINE-1 sequence to become hypomethylated.

### Distinguishing between tumor and healthy tissue by locus-specific LINE-1 methylation

The detailed and sensitive analysis of L1Hs locus-specific DNA methylation in several tumors presented here has allowed us to study its potential for distinguishing tumor from healthy tissue. The majority of samples (90%) carry significant methylation changes in at least one LINE-1 locus. Therefore, an approach for detection of tumors based on LINE-1 locus-specific methylation could be quite effective, considering that the number of analyzed loci could be extended by an appropriate multiplexing strategy.

The number of loci affected per sample varied between the cancer types, with the highest number of affected loci in bladder cancers and the lowest in colon and prostate cancers. These findings agree well with previous reports on global LINE-1 hypomethylation. For instance, global LINE-1 hypomethylation in prostate cancer has been observed to be relatively limited in many primary tumors, aggravating mostly in metastatic cases, whereas global LINE-1 hypomethylation is highly prevalent throughout all stages of bladder cancer [20,33,34].

The utility of measuring DNA hypomethylation and, specifically, LINE-1 hypomethylation in blood to detect cancers and to gauge individual cancer risk is intensely debated. Obviously, different studies reported different findings related to the significance of the LINE-1 hypomethylation in peripheral blood of cancer patients; the reason for this could be related to differences in cancer types (i.e. tissue affected) as well as stages of cancer, to the methods used to detect the methylation differences, to the compatibility of the control group with the studied cases and/or to the differences in environmental factors and living conditions in different patient cohorts. Based on a meta-analysis, Woo and Kim [2] concluded that the overall cancer risk in the studies groups with the lowest LINE-1 DNA methylation levels in peripheral blood were significantly higher when compared to the studies with the highest methylation levels. However, they were cautious and mentioned that different experimental procedure could have confounded the comparisons. Brennan and Flanagan have recently concluded that global LINE-1 hypomethylation in blood may not suit these purposes well [14], Likewise, Barchitta *et al*., [11] in a more recent meta-analysis, concluded that significant methylation differences between cancer and healthy samples were widespread for tissues but not reliably detectable in peripheral blood. In the present study, we likewise did not observe a significant association between the presence of cancer and changes in global LINE-1 methylation in blood. In fact, in the cancer tissue samples, we observed that the presence of hypermethylation as well as hypomethylation at various individual LINE-1 elements tends to eliminate the methylation difference between tumor and healthy tissues. The same effect may also pertain to DNA methylation changes in blood. As argued above, an analysis using methylation levels at several appropriately selected individual LINE-1 loci may be most likely to succeed (even for blood samples). Such an analysis might allow identifying individuals with cancer or at high risk rather than just classifying healthy and unhealthy individuals into two large overlapping groups. Studies of larger number of loci, which are particularly strongly demethylated in different tumors and in patient blood, are required to improve the sensitivity and specificity of such an assay.

In the present study, no significant improvement in the diagnostic power was gained by using any single locus over the degenerate LINE-1 methylation assay. Most likely, the optimal approach to diagnostic application may consist in combining methylation at several carefully evaluated individual loci into a single score. To this end, three limitations of this study must be overcome. First, a larger number of samples should be used for each tumor type. Second, larger numbers of normal tissues derived from healthy persons should be included, and third, a larger number of individual LINE-1 loci should be simultaneously investigated. Nevertheless, our study strongly reconfirmed the clinical diagnostic benefit of using LINE-1 methylation as a biomarker to identify carcinogenic tissues and supports caution about its use in blood samples in line with recent meta-analyses [11]. Moreover, we provide evidence that locus-specific LINE-1 can also be used to classify healthy from non-healthy tissues albeit the correct predictive markers for different type of tumors has still to be identified. Additionally, our study has identified specific CpG sites within the L1 promoter consensus sequence that were more sensitive for hypomethylation and thus could provide better targeting regions for clinical tests.

Recently, whole-genome sequencing of cancer genomes has revealed several instances of somatic retrotranspositions testifying to the activation of LINE-1 retroelements in human cancers [35]. Likewise, work from our group [19,20] and others [13,18] have documented an increased overall level of LINE-1 transcripts in various cancer types. Nevertheless, the exact relationship between LINE-1 hypomethylation and re-expression in cancer remains unclear [13]. Among the 11 full-length L1Hs elements studied by us, only few were detectably expressed in cancer tissues, despite often considerable hypomethylation. As individual retrotransposition-competent LINE-1 elements are thought to number well below 100, this could mean that only a small fraction of them might become overexpressed in each cancer type. Accordingly, previous studies [36,37] have reported expression of LINE-1 in a broad range of normal tissues that appeared to be dominated by a few elements in each one. We observed that those loci becoming induced in cancers already presented with detectable expression in normal tissue, suggesting that expression of individual LINE-1 loci in cancer might be favored by an already partially active state in the normal precursor tissue. Therefore, the individual LINE-1 elements already partially active in specific tissues might be the ones reacting most strongly to hypomethylation.

## Conclusions

In summary, we present the first detailed methylation profiling of individual specific LINE-1 loci in tissues from common human cancers. Our data reveal several characteristics of the methylation changes at full-length LINE-1 sequences associated with tumor development. In particular, hypomethylation patterns at a given individual locus are not completely random, as defined CpGs are more prone to hypomethylation. These could act as seeds from which abnormal methylation spreads to the neighboring sequence.

## Methods

### Patients and samples

Twenty-four bladder and 20 prostate cancer samples were obtained from the Department of Urology, Heinrich Heine University (HHU), Düsseldorf, Germany. Twenty-one colon cancer, 22 stomach cancer, and 20 pancreatic cancer samples were obtained from the Institute for Digestive Research, Lithuanian University of Health Sciences, Vilnius. Paired healthy and cancerous fresh frozen tissues were available for colon, bladder, prostate, and stomach tumors. The study was approved by the Ethics Committee of the Medical Faculty of the HHU and the Lithuanian University Bioethics Committee (approval numbers 1352 and BE-2-17, respectively). Written informed consent was obtained from all patients. The clinical-pathological characteristics of the tumors based on the International Union Against Cancer TNM classification (seventh edition) are summarized in Additional file 17. Patients had neither undergone chemotherapy nor radiotherapy prior to surgery. Additionally, normal tissues from individuals without cancer were available for colon (11 males and 11 females), pancreas (five males and five females), and stomach (11 males and 11 females). Blood samples from the pancreatic, stomach, and colon cancer patients were taken immediately prior to surgery. Control healthy blood samples (34 males and 24 females) were obtained from the Blood Donation Department at the Institute of Experimental Hematology and Transfusion Medicine, University Clinics Bonn, Germany. Samples from patients with untypical X-chromosome status were excluded as previously described [15].

### DNA methylation analysis

#### Global LINE-1 methylation

The global LINE-1 assay SIRPH was essentially performed as previously described [16,38,39]. For SIRPH detection of LINE-1 methylation, SN-8 and SN-9 primers were used which interrogate two CpG sites in the L1Hs consensus sequence as previously described [16].

#### Locus-specific LINE-1 methylation analysis

Pyrosequencing and amplification of specific LINE-1 loci were done as previously described by Singer *et al*. [15]. For sequence information, genomic positions, and primers used for amplifications, see Additional file 1. These loci were chosen based on their high homology to the consensus sequence (L1Hs; NCBI accession number: X58075). From three X-linked loci, namely L1X4, L1X5, and L1X6, data were generated using two different pyrosequencing primers, which were designated as L1X4a and L1X4b, L1X5a and L1X5b, and L1X6a and L1X6b.

#### Next-generation sequencing

Specific LINE-1 amplification products were subjected to next-generation sequencing to reveal the methylation status of individual DNA molecules covering all CpG sites in the LINE-1 5′ region. Briefly, purified and equimolarly pooled amplicons (lengths 190 to 430 bp) were used as input for preparation of a 454 Genome Sequencer FLX (Roche, Mannheim, Germany) sequencing library following the manufacturer’s protocol using bar-coded rapid library adaptors (454 Sequencing System Guidelines for Amplicon Experimental Design, May 2011). The resulting libraries were titrated and used as input for the emulsion PCR (GS FLX Titanium emPCR Kit Lib-L) with 32 copies per bead. The beads were sequenced in two regions (four-region gasket) of a PicoTiterPlate by GS FLX Titanium chemistry (Roche, Mannheim, Germany).

#### Expression analysis of individual and total LINE-1

Total RNA was extracted from powdered tissues as described previously [20]. Of note, RNA extraction involved acid phenol extraction followed by column purification to minimize DNA contamination. Synthesis of complementary DNA was performed using the QuantiTect Reverse Transcription Kit (Qiagen, Hilden, Germany) according to the manufacturer’s protocol, including an extra DNA removal step as recommended by the supplier. qRT-PCR was performed on a 7500 Fast Real-Time PCR System (Applied Biosystems, Carlsbad, CA, USA) using QuantiTect SYBR Green PCR Kit (Qiagen) with specifically designed primers for both specific LINE-1s and total LINE-1 (Additional file 1). qRT-PCR data were adjusted to TATA-box-binding protein (TBP) mRNA levels. All measurements were performed at least in duplicates; technical assay variance was less than 10%. Relative expression was calculated by a modified ΔΔCt method published by Pfaffl [40].

#### Modeling nucleosome structures based on the crystal structure of Widom sequence

*In silico* structural analysis was done for a consensus LINE sequence derived from the 11 individual sequences analyzed in this study. Nucleosome positioning predictions were done using three methods: 1) a freely available web server (SymCurv at http://genome.crg.es/cgi-bin/SymCurv/SymCurv_borinot.pl/accessed on 31/08/2013) which works on the basis of DNA curvature, 2) experimental nucleosome probability data available at the University of California, Santa Cruz (UCSC), and 3) an improvised method based on sequential similarity to the nucleosome core particle (NCP)-601L Widom sequence.

For the first approach, the online method SymCurv uses a greedy algorithm to parse a sequence fitted on the basis of symmetry constraints into nucleosome-bound and nucleosome-free segments. For this approach, we chose the nucleosome DNA prediction that overlapped in most LINE-1 sequences (nine showed the same DNA occupancy prediction). For the second approach, we downloaded experimental nucleosomal probability data from UCSC and defined one nucleosome position of interest within the consensus LINE sequence based on two consecutive crests and troughs for the multiple curves which were spaced approximately 146 bp apart, i.e., one bound nucleosomal DNA length. For the last nucleosome positioning method, we aligned the consensus LINE sequence with the palindromic NCP-601L Widom sequence using a disable gap weighting (speed oriented) method implemented by ClustalWS in the Jalview 2.7 Java-based multiple sequence alignment editor implementing the Java Bioinformatics Analysis Web Services system (JABAWS). The resulting alignment was further manually curated to remove any remaining gaps.

The three methods resulted in three different, partly overlapping sequences covering varied numbers of CpGs of interest. A DNA model was generated for all these sequences using the basic step parameter from the NCP-601L nucleosomal crystal structure file (PDB ID: 3UT9) on the 3-D DNA web server. The generated models were then docked onto an octamer histone structure stripped of its DNA (source: NCP-601L nucleosomal structure PDB ID: 3UT9) and other heteroatoms. Docking was performed on the ZDOCK server (http://zdock.umassmed.edu/, accessed on 1 January 2013) using default positions. The docked models were relaxed and refined by a short 500 ps solvated simulation. The lowest energy structure was used as the final model for the analysis of all CpGs of interest. The CpGs within each sequence were analyzed in terms of exposure of cytosine prone to methylation, positioning/rotational orientation with respect to the histones and in the context of their major and minor groove location. All structural calculations and image rendering were done with YASARA version 13.3.26.

#### Statistical analysis and data processing

Statistical analysis was done using the Prism software (GraphPad Prism version 5.0f, GraphPad Software, San Diego California USA, www.graphpad.com) or the SAS software (SAS for Windows, version 9.1.; SAS Institute Inc., Cary, NC, USA). The next-generation sequencing data were analyzed using the Geneious software version 6.03 (Biomatters Ltd, Australia). Principle component analysis, heat maps, and depiction of tissue-specific differences were done using the Qlucore omics explorer version 2.3 (Qlucore AB, Sweden). Methylation entropy was calculated using a sliding window of four CpGs according to Xie *et al*. [41].

A Wilcoxon signed rank test was applied to identify markers showing statistically significant differences between healthy and tumor samples. In case of blood samples, a Kruskal-Wallis test was used, which aims to detect markers varying significantly between different cancer types and healthy controls. *P* values were corrected for multiple testing using Benjamini and Hochberg’s control of false discovery rate (FDR). Markers with FDR <5% were combined in a multivariate logistic regression model, giving rise to a probability for each sample to be tumor.

The class separation ability of the multivariate logistic regression model was measured via the area under ROC curve (AUC; 1 = perfect classification, 0.5 = chance level). The AUC is a measure which does not rely on a pre-defined cutoff for class probabilities *π*.

## Additional files

Additional file 1:
**Table sequence and primer information.**
Additional file 2:
**Comparison of LINE-1 methylation of different healthy tissues.** Vertical scatter plots of methylation values at different LINE-1 loci in healthy tissues. The number of samples analyzed for each tissue at each locus is given above the tissue name abbreviations. *P* values of Krustal-Wallis test for one-way ANOVA are shown on each plot; the significant ones are labeled in red. Male and female samples are represented by red and blue dots, respectively. Horizontal lines with stars represent the results of the Dunn multiple comparison test (for tissue-tissue comparisons), whereby one, two, and three stars correspond to *p* values <0.05, ≤ 0.01, and ≤ 0.001, respectively. Ba: bladder, Pr: prostate, St: stomach, Co: colon, Pa: pancreas, Bl: blood. Horizontal lines on each group of data represent the mean values and the standard deviations. Additional file 3:
**Comparison between the histological normal tissue neighboring the tumor tissue (tissue) and non-tumor tissue from healthy individuals (controls).** Only some loci are selected for colon and stomach tissues. Mann-Whitney test *p* values are shown above each comparison. None of the comparisons were statistically significant. The number of samples analyzed for each tissue at each locus is given above the type of samples (control or tissue). Additional file 4:
**Pair-wise comparisons of paired tissue (normal vs. cancer) samples of (A) bladder, (B) stomach, (C) colon, (D) prostate, and (E) non-paired sample of pancreatic cancer.** Both methylation data from locus-specific LINE-1 (14 loci) and the global LINE-1 (two) assays are shown; male and female data are analyzed separately. Mann-Whitney test *p* values are indicated under the name of the locus; a transparent pink box highlights loci showing statistical significance. However, after correction for multiple testing (significance threshold of *p* < 0.00313) only L1X6b in stomach and L1C10 in colon remained significant. The number of samples analyzed for each healthy or tumor group at each loci is given below the scatter plot. Additional file 5:
**Simple statistics on distribution of affected loci (A) Distribution and number of hypo/hyper-methylated LINE-1 loci in each patient (sorted in increasing numbers of affected loci).** (B) Histograms showing the number of tumor tissues affected at a given number of loci. Hyper and hypo- methylated are defined as methylation differences between paired tissues of >10% and <10%, respectively. Additional file 6:
**Separation ability of the multivariate logistic regression model as measured by the area under the curve.** Only significant markers are included. Each of the blue or red filled circles corresponds to tumor and healthy tissues, respectively; one blue and one red circle in a given vertical line correspond to one-paired sample. AUC: area under the curve, vertical descendent arrows indicate the paired samples that failed to separate in this model (i.e., the red circle corresponding to healthy tissue is above the tumor tissue). The number of samples included in the analysis is shown to the left of the figures. Additional file 7:
**Correlation of clinical characteristics and methylation levels.**
Additional file 8:
**Expression analysis of individual LINE-1 elements in prostate cancer tissues.** LINE-1_C2, LINE-1_C11, and LINE-1_X4 RNA levels were measured by qRT-PCR in 12 benign and 47 prostate cancer tissues. RNA levels were each normalized to TBP and standardized to the median RNA level of benign tissues set as 1. *P* values were calculated by the Mann-Whitney *U*-test. A number of samples are indicated below the box plots. Additional file 9:
**Comparisons of individual blood samples of tumor patients and healthy donors.** Results of locus-specific LINEs methylation (11 loci) as well as global LINE-1 methylation (two assays, SN8 and SN9) are shown; male and female data are represented separately. Mann-Whitney test *p* values are shown in red for statistically significant comparisons. The number of samples analyzed for each group at each locus is given below the scatter plot. Additional file 10:
**ROC curves for blood methylation values for colon, pancreas, and stomach cancer patients.** Green lines are for specific loci that survived the linear regression analysis (L1X8, L1X8-L1X4A, and L1X4A-L1X8 for colon, pancreas, and stomach, respectively), while orange ones are for the global assay of SN8 and SN9. The areas under the curves (AUC) are given for each of the curves; results were derived from 59 blood samples from healthy individuals and 21, 22, and 20 blood samples from colon, stomach, and pancreatic cancer patients. Additional file 11:
**Summary heat maps of massive parallel sequencing of five specific autosomal and six specific X-linked LINE-1 loci from control blood (corresponding to a pool of five healthy individuals) and selected individual blood samples of tumor patients.** Every line in the heat map corresponds to a sequence originating from an individual PCR DNA molecule, each colon represent a CpG site at the promoter of the specific LINE-1. Below the heat maps, the average of the methylation of all sequences and all CpG sites are given followed by the average methylation entropy. The numbers of sequences that construct the heat map are shown at the outer upper left side. A graph representing the methylation average at every CpG site of tumor tissue (in red) and histologically normal tissue (in green) is shown above each heat map. *P* values (Wilcoxon test) for differences of methylation are also shown. Above that, a diagram depicting the entropy (tumor in red, healthy in green) in sliding windows of four CpGs is shown with the corresponding *p* values (Wilcoxon test). Horizontal red bars represent the regions studied by pyrosequencing. Additional file 12:
**The hypo- and hypermethylated hotspots in different LINE-1 loci: (A) for autosomal loci and (B) for X-linked loci.** The upper panel shows the average methylation for all six sequenced patients at all CpG for a given locus, the vertical blue and green arrows correspond to the relatively hyper- and hypomethylated peaks. The middle panel shows the distribution of the individual values for the six patients, red and gray horizontal lines represent the mean and standard deviations; significant differences based on non-parametric *t*-test (Mann-Whitney test) are indicated by horizontal bars and stars (one and two stars correspond to *p* < 0.05 and *p* <0.001, respectively), at the top of the graph (calculated for two neighboring CpG sites (*n* and *n* + 1) and the next CpG site (*n* and *n* + 2)). The lower panel shows the heat map of combined sequences from all six patients; the significant chi square comparisons of consecutive CpG sites are indicated by asterisks at the lower part (one asterisk represents significance after Bonferroni correction for multiple testing). Additional file 13:
**Average of methylation levels of six NGS sequenced tumor samples at individual CpGs: (A) per patient and (B) per region.** Autosomal and X-linked loci were analyzed separately. Additional file 14:
**Inter-sample correlation between average CpG methylation at a given L1 sequence: (A) X-linked loci and (B) autosomal loci.** All samples studied by massive parallel sequencing were compared to each others. The rho Spearman correlation (lower left triangle) and the *p* values (upper right triangle) are represented by heat map boxes, one for each region. Additional file 15:
**Summary representation of massive parallel sequencing results of selected CpG islands in breast and colon cancer reported by Varley** ***et al*** **.** [21]. The left, middle, and right part represent breast, colon, and correlation data, respectively. Heat maps representing combinations of all samples in one group are shown in yellow (unmethylated) and blue (methylated), whereby each column represents one CpG site and each line represent one sequence reads. The average of both the tumor and the healthy samples are shown to the right of the graph. Horizontal red arrows indicate the regions with the highest inter-sample correlations indicating a specific methylation pattern present across different samples, different tissues, and different disease status. Additional file 16:
**Inter-locus correlations of all LINE-1 specific loci (based on pyrosequencing data) and the global LINE-1 assay (SN8 and SN9 based on SIRPH data).** Inter-locus correlation based on the average within one region was separately performed for healthy tissue samples and tumors and for male and female. In the right upper and the low left triangles, *p* values and Spearman rho values are represented in a heat map. Only significant *p* value correlations are depicted. The significant correlations after Bonferroni corrections for multiple testing are highlighted in black squares. The number of sample used in each group is given above the correlation boxes. Additional file 17:
**Clinical characteristics of the tumor samples for the (A) bladder, (B) colon, (C) pancreas, (D) prostate, and (E) stomach.**
