# Supplementary figures and images for: Inter-locus as well as intra-locus heterogeneity in LINE-1 promoter methylation in common human cancers suggests selective demethylation pressure at specific CpGs
Source: Clin Epigenetics. 2015 Mar 1;7(1):17. doi: 10.1186/s13148-015-0051-y (PMC4367886; doi:10.1186/s13148-015-0051-y)

# Histological normal Tissue and Healthy Blood

Locus Specific LINE-1s

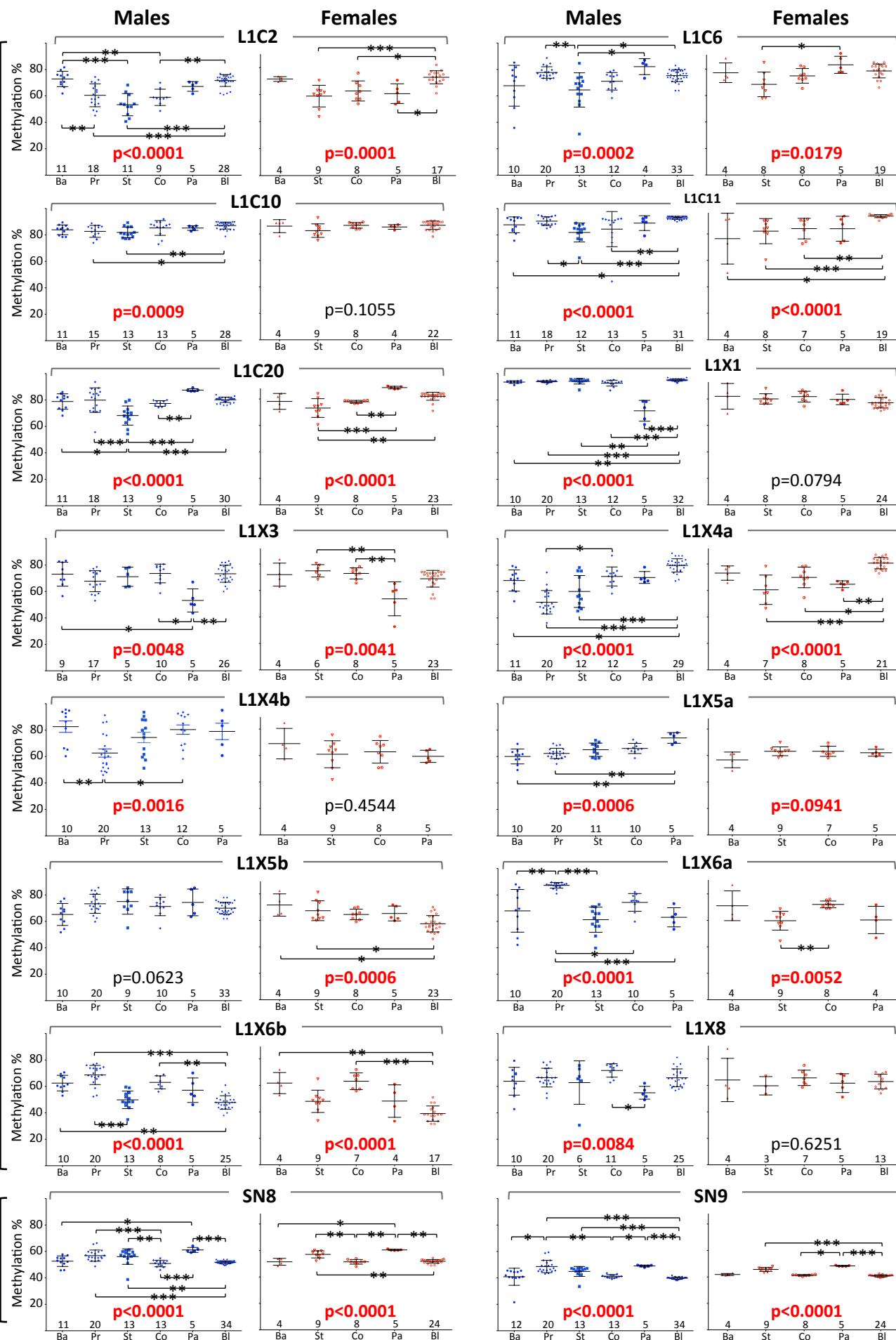

Global

Supplement: Additional file 2: — Comparison of LINE-1 methylation of different healthy tissues. Vertical scatter plots of methylation values at different LINE-1 loci in healthy tissues. The number of samples analyzed for each tissue at each locus is given above the tissue name abbreviations. P values of Krustal-Wallis test for one-way ANOVA are shown on each plot; the significant ones are labeled in red. Male and female samples are represented by red and blue dots, respectively. Horizontal lines with stars represent the results of the Dunn multiple comparison test (for tissue-tissue comparisons), whereby one, two, and three stars correspond to p values <0.05, ≤ 0.01, and ≤ 0.001, respectively. Ba: bladder, Pr: prostate, St: stomach, Co: colon, Pa: pancreas, Bl: blood. Horizontal lines on each group of data represent the mean values and the standard deviations. [file 13148_2015_51_MOESM2_ESM.pdf]

## Colon

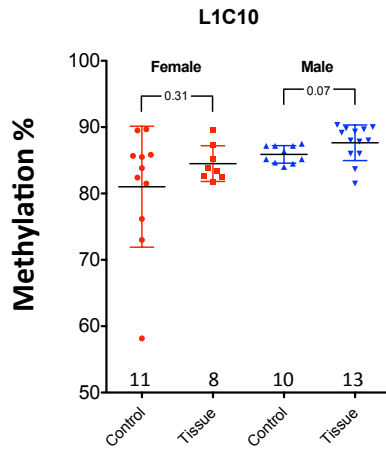

## Stomach

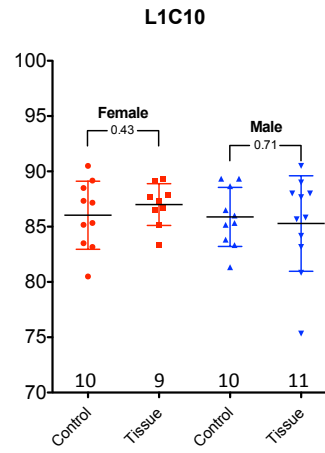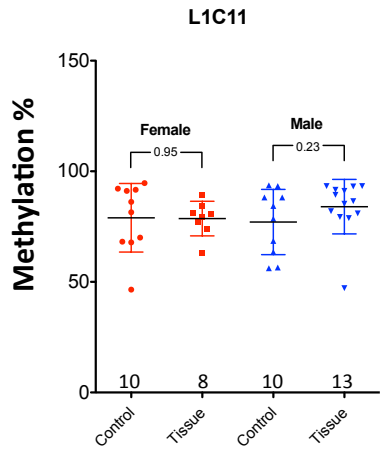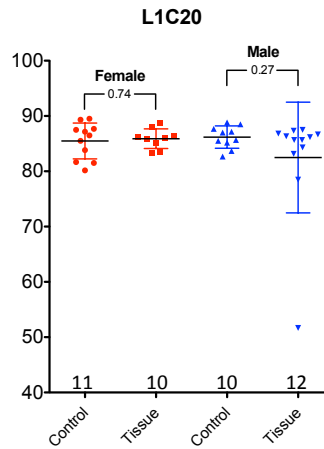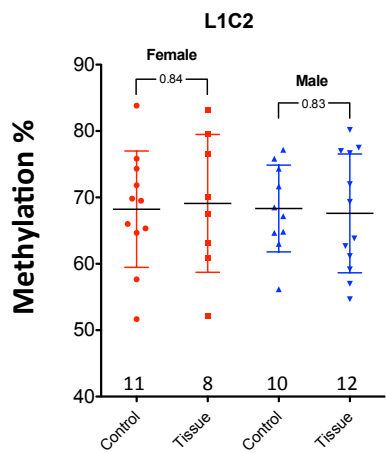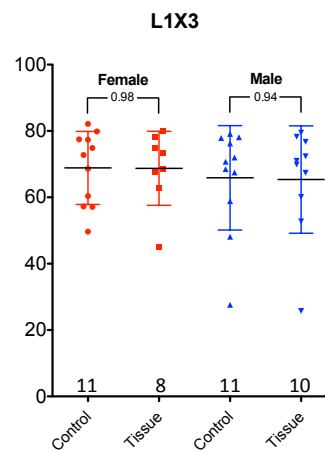

Supplement: Additional file 3: — Comparison between the histological normal tissue neighboring the tumor tissue (tissue) and non-tumor tissue from healthy individuals (controls). Only some loci are selected for colon and stomach tissues. Mann-Whitney test p values are shown above each comparison. None of the comparisons were statistically significant. The number of samples analyzed for each tissue at each locus is given above the type of samples (control or tissue). [file 13148_2015_51_MOESM3_ESM.pdf]

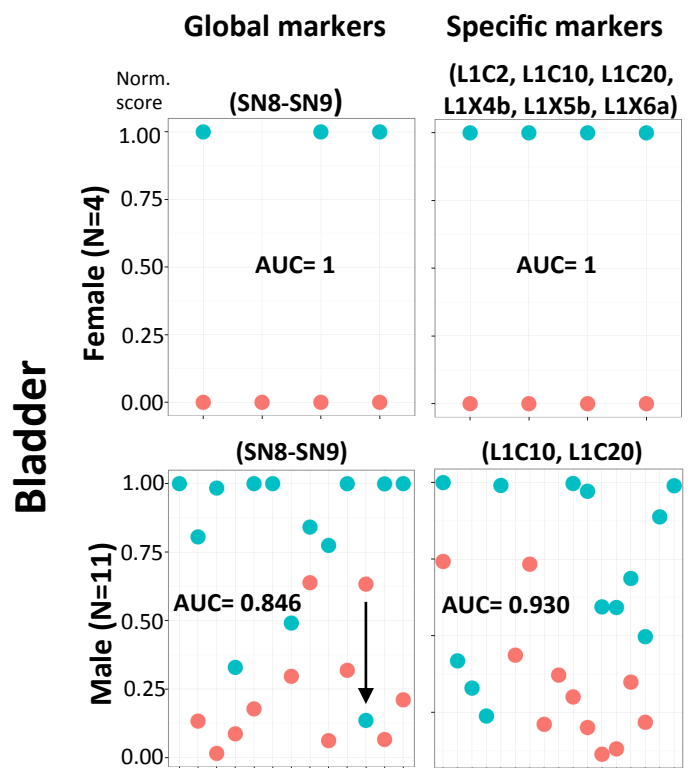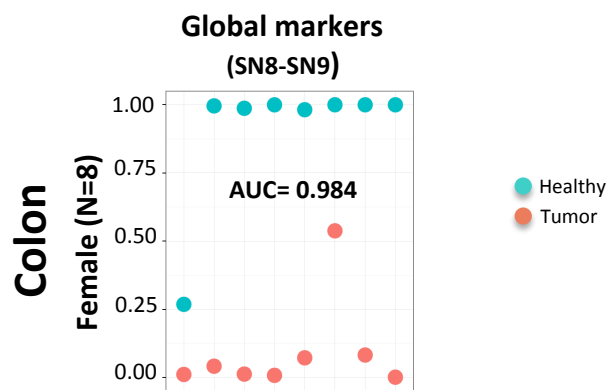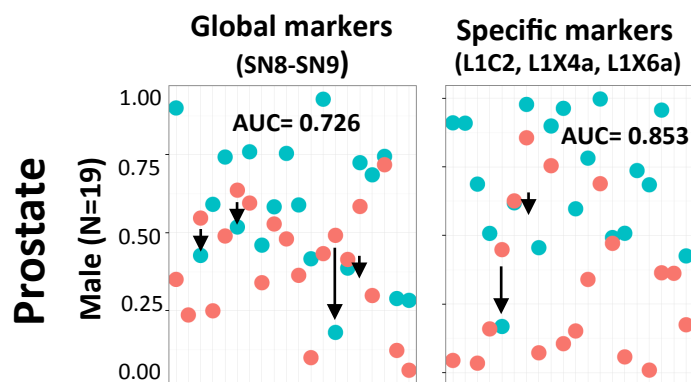

Supplement: Additional file 6: — Separation ability of the multivariate logistic regression model as measured by the area under the curve. Only significant markers are included. Each of the blue or red filled circles corresponds to tumor and healthy tissues, respectively; one blue and one red circle in a given vertical line correspond to one-paired sample. AUC: area under the curve, vertical descendent arrows indicate the paired samples that failed to separate in this model (i.e., the red circle corresponding to healthy tissue is above the tumor tissue). The number of samples included in the analysis is shown to the left of the figures. [file 13148_2015_51_MOESM6_ESM.pdf]

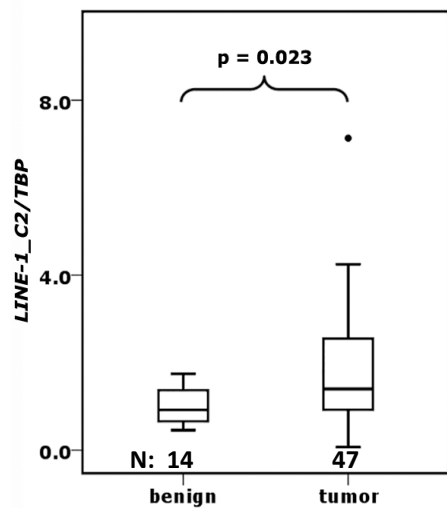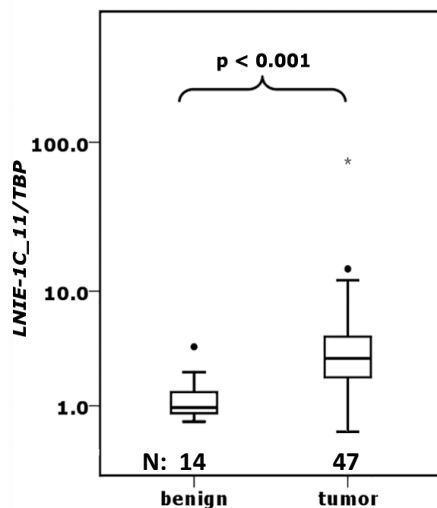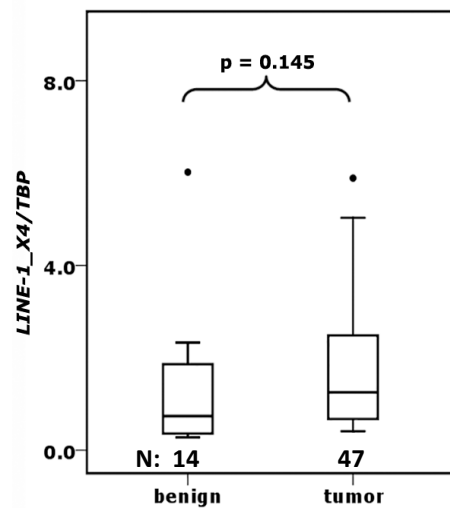

Supplement: Additional file 8: — Expression analysis of individual LINE-1 elements in prostate cancer tissues. LINE-1_C2, LINE-1_C11, and LINE-1_X4 RNA levels were measured by qRT-PCR in 12 benign and 47 prostate cancer tissues. RNA levels were each normalized to TBP and standardized to the median RNA level of benign tissues set as 1. P values were calculated by the Mann-Whitney U-test. A number of samples are indicated below the box plots. [file 13148_2015_51_MOESM8_ESM.pdf]

# Blood (from tumor patients and healthy individuals)

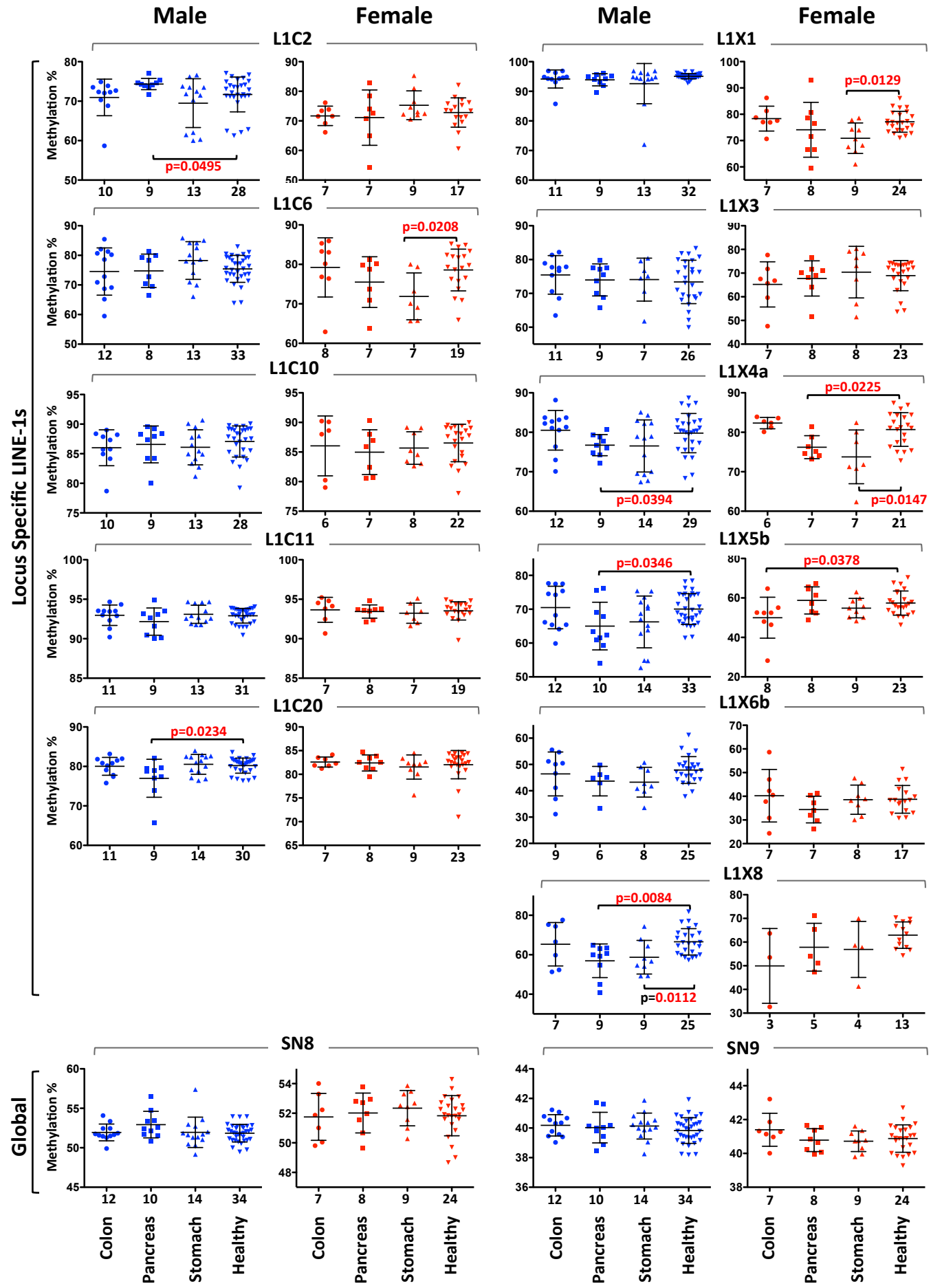

Supplement: Additional file 9: — Comparisons of individual blood samples of tumor patients and healthy donors. Results of locus-specific LINEs methylation (11 loci) as well as global LINE-1 methylation (two assays, SN8 and SN9) are shown; male and female data are represented separately. Mann-Whitney test p values are shown in red for statistically significant comparisons. The number of samples analyzed for each group at each locus is given below the scatter plot. [file 13148_2015_51_MOESM9_ESM.pdf]

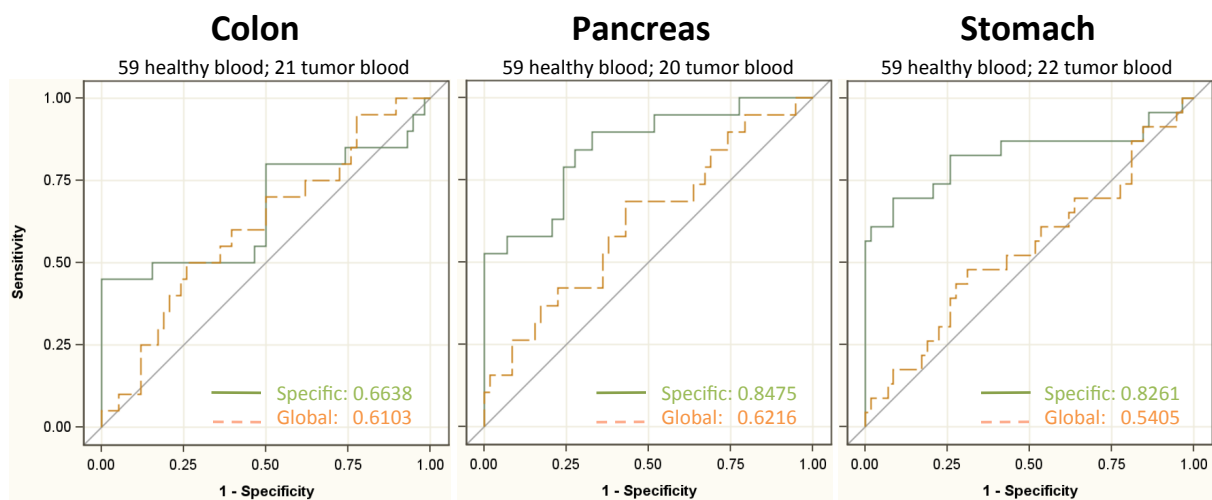

Supplement: Additional file 10: — ROC curves for blood methylation values for colon, pancreas, and stomach cancer patients. Green lines are for specific loci that survived the linear regression analysis (L1X8, L1X8-L1X4A, and L1X4A-L1X8 for colon, pancreas, and stomach, respectively), while orange ones are for the global assay of SN8 and SN9. The areas under the curves (AUC) are given for each of the curves; results were derived from 59 blood samples from healthy individuals and 21, 22, and 20 blood samples from colon, stomach, and pancreatic cancer patients. [file 13148_2015_51_MOESM10_ESM.pdf]

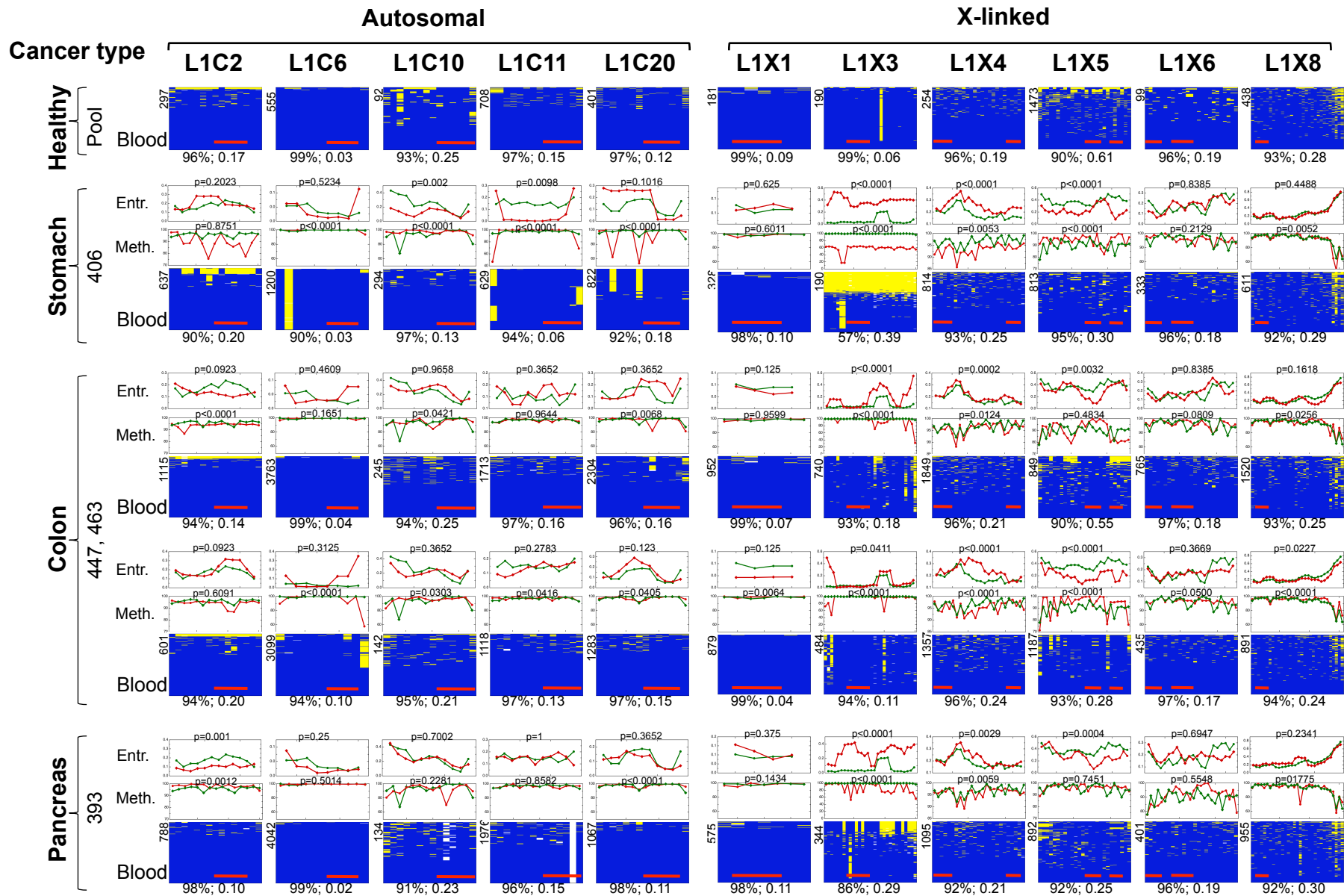

Supplement: Additional file 11: — Summary heat maps of massive parallel sequencing of five specific autosomal and six specific X-linked LINE-1 loci from control blood (corresponding to a pool of five healthy individuals) and selected individual blood samples of tumor patients. Every line in the heat map corresponds to a sequence originating from an individual PCR DNA molecule, each colon represent a CpG site at the promoter of the specific LINE-1. Below the heat maps, the average of the methylation of all sequences and all CpG sites are given followed by the average methylation entropy. The numbers of sequences that construct the heat map are shown at the outer upper left side. A graph representing the methylation average at every CpG site of tumor tissue (in red) and histologically normal tissue (in green) is shown above each heat map. P values (Wilcoxon test) for differences of methylation are also shown. Above that, a diagram depicting the entropy (tumor in red, healthy in green) in sliding windows of four CpGs is shown with the corresponding p values (Wilcoxon test). Horizontal red bars represent the regions studied by pyrosequencing. [file 13148_2015_51_MOESM11_ESM.pdf]

A)

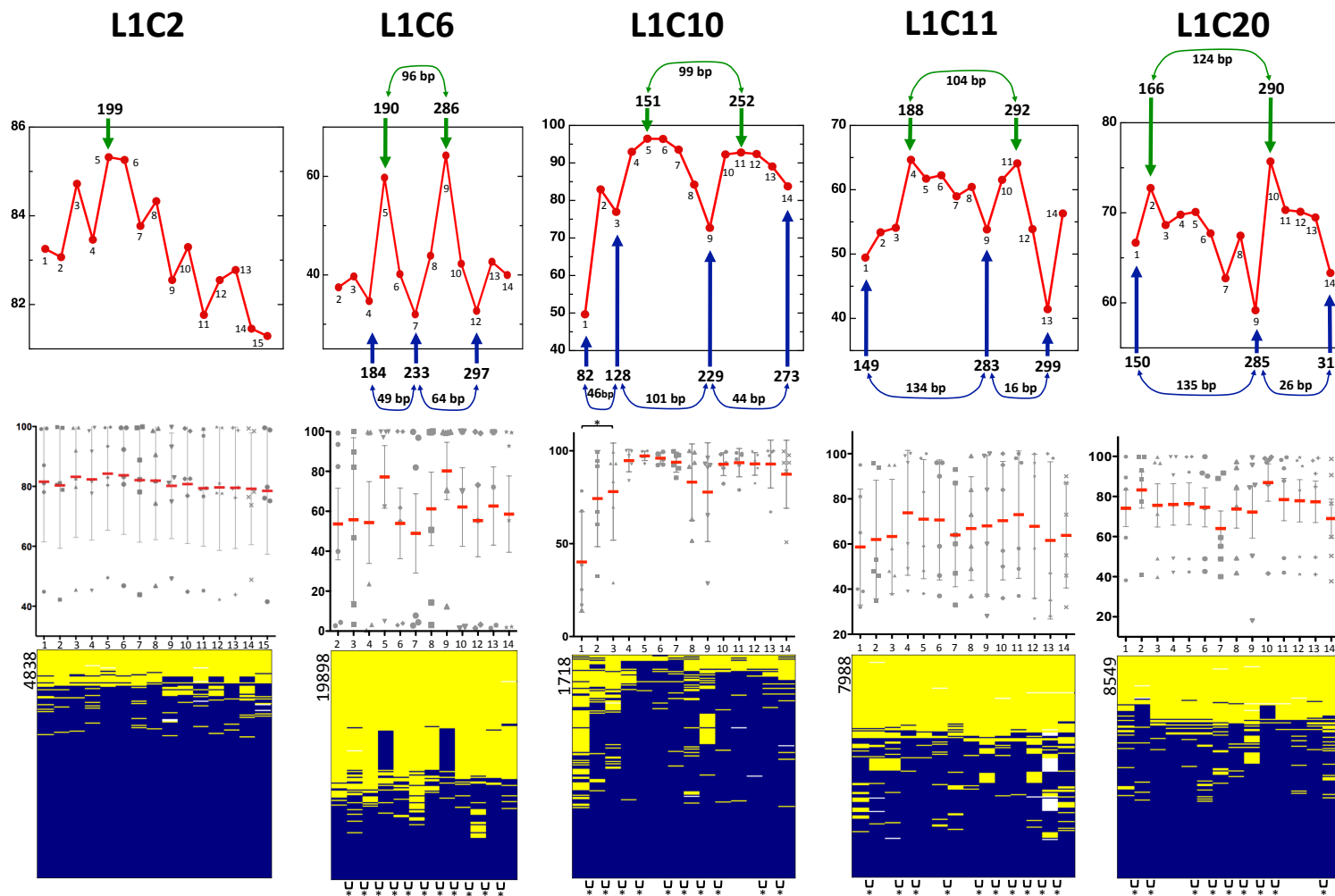

B)

L1X1

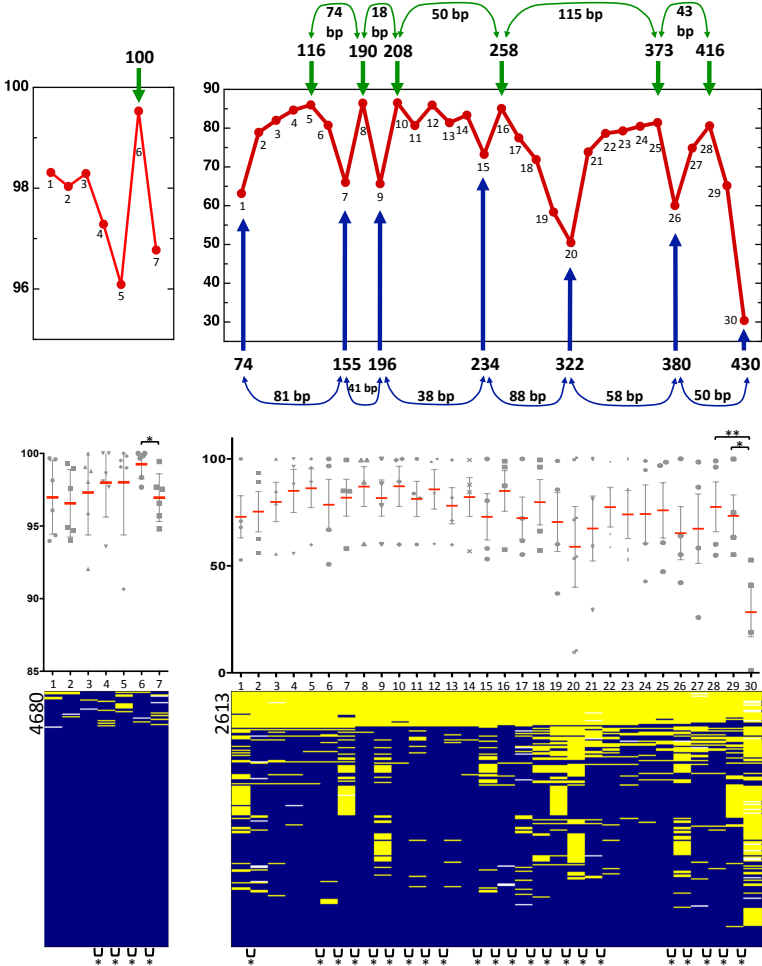

L1X3

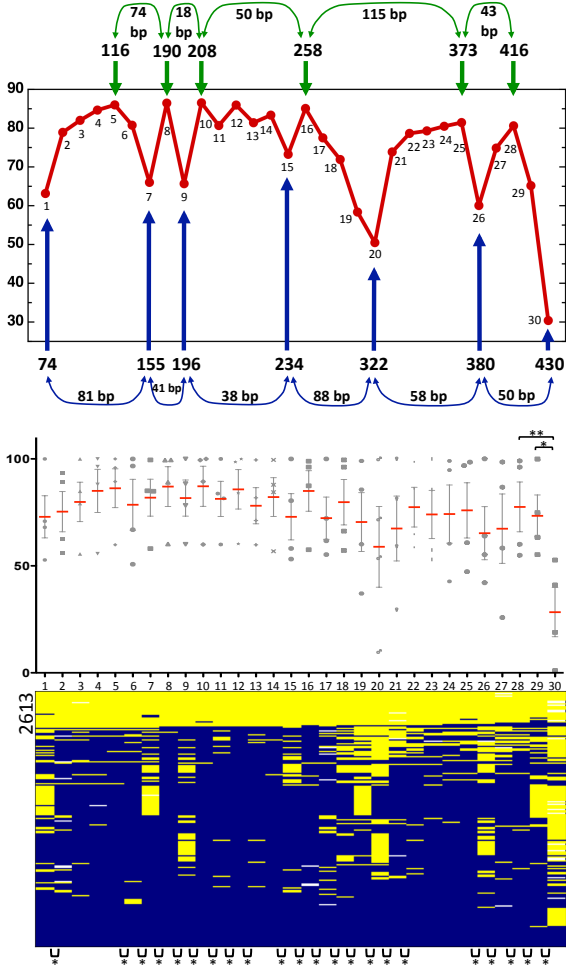

L1X4

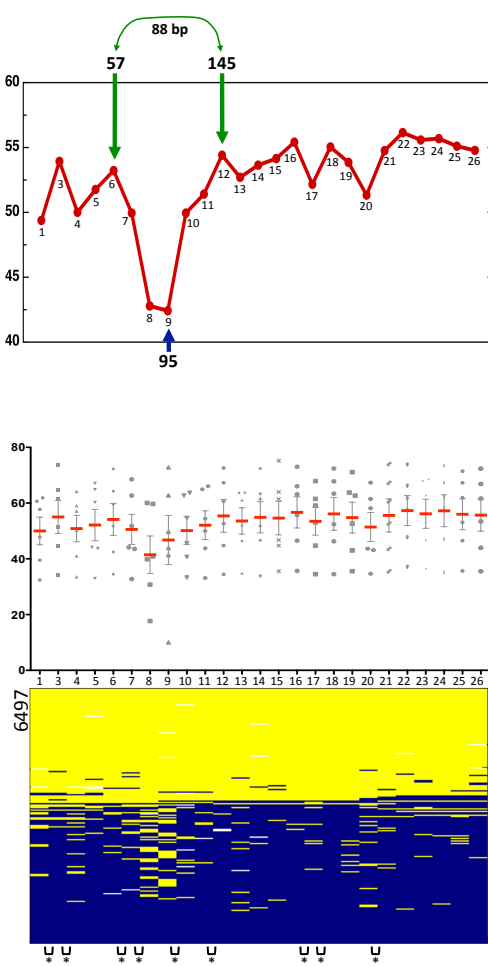

L1X5

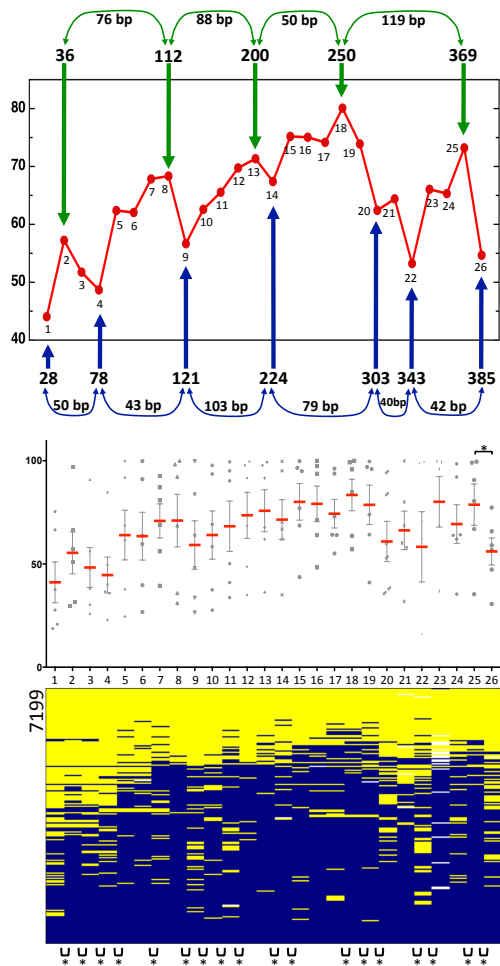

B) Continued

L1X6

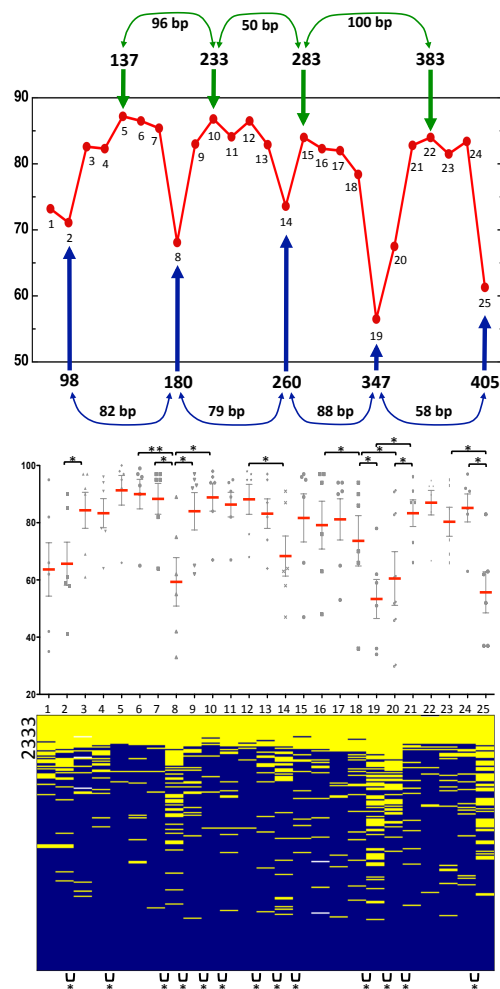

L1X8

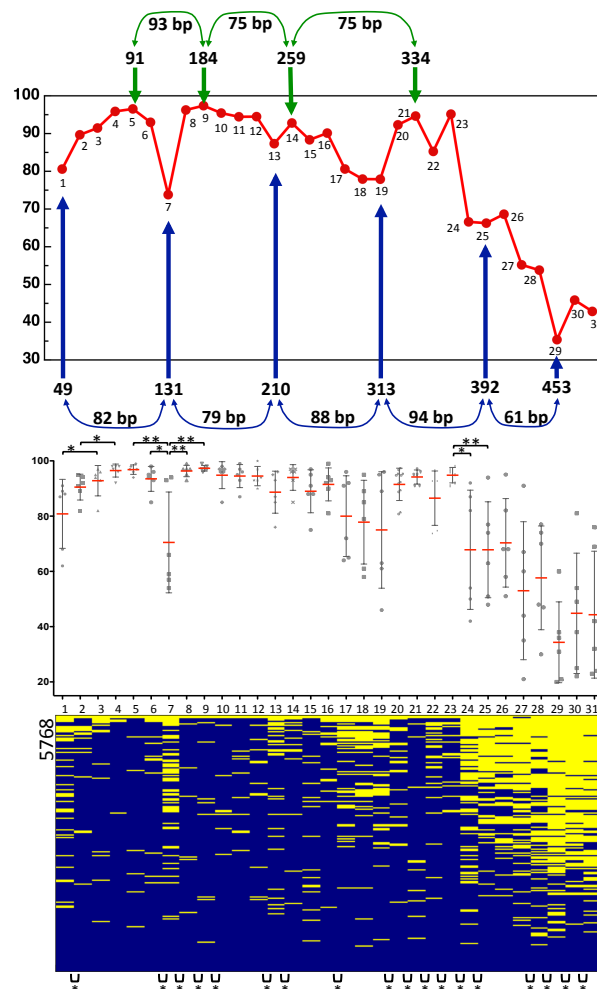

Supplement: Additional file 12: — The hypo- and hypermethylated hotspots in different LINE-1 loci: (A) for autosomal loci and (B) for X-linked loci. The upper panel shows the average methylation for all six sequenced patients at all CpG for a given locus, the vertical blue and green arrows correspond to the relatively hyper- and hypomethylated peaks. The middle panel shows the distribution of the individual values for the six patients, red and gray horizontal lines represent the mean and standard deviations; significant differences based on non-parametric t-test (Mann-Whitney test) are indicated by horizontal bars and stars (one and two stars correspond to p < 0.05 and p <0.001, respectively), at the top of the graph (calculated for two neighboring CpG sites (n and n + 1) and the next CpG site (n and n + 2)). The lower panel shows the heat map of combined sequences from all six patients; the significant chi square comparisons of consecutive CpG sites are indicated by asterisks at the lower part (one asterisk represents significance after Bonferroni correction for multiple testing). [file 13148_2015_51_MOESM12_ESM.pdf]

**A) Per Patient**

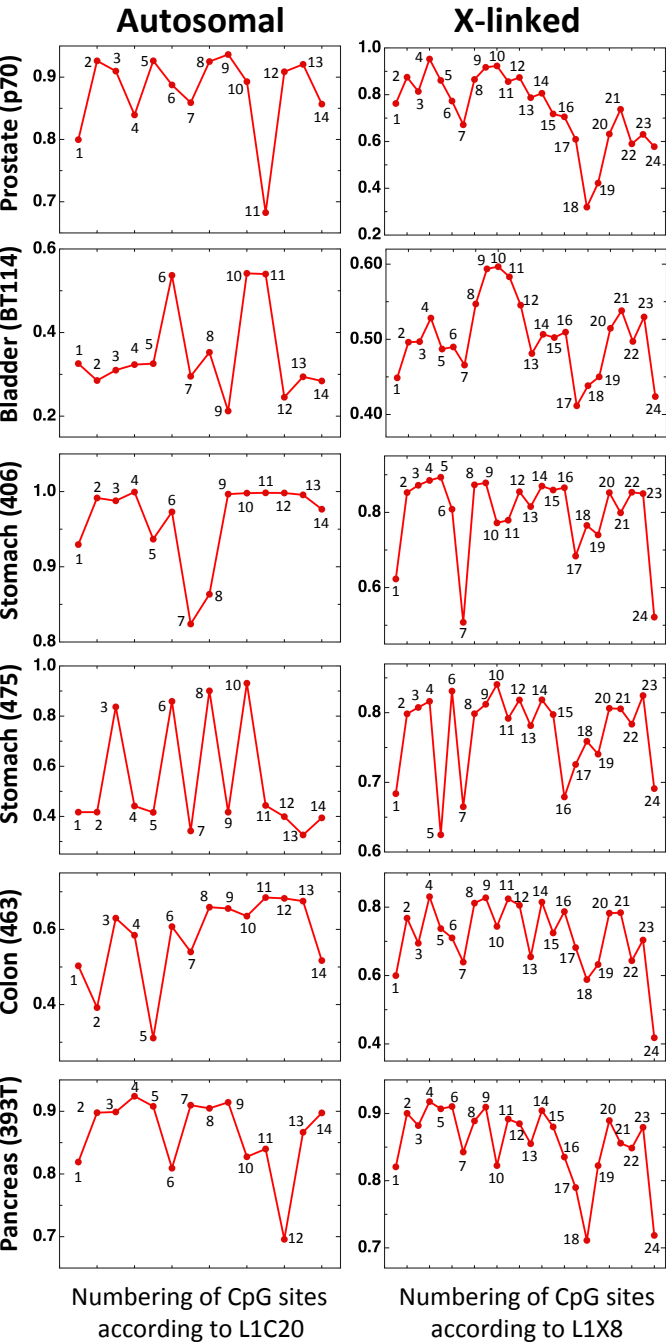

**B) Per Region**

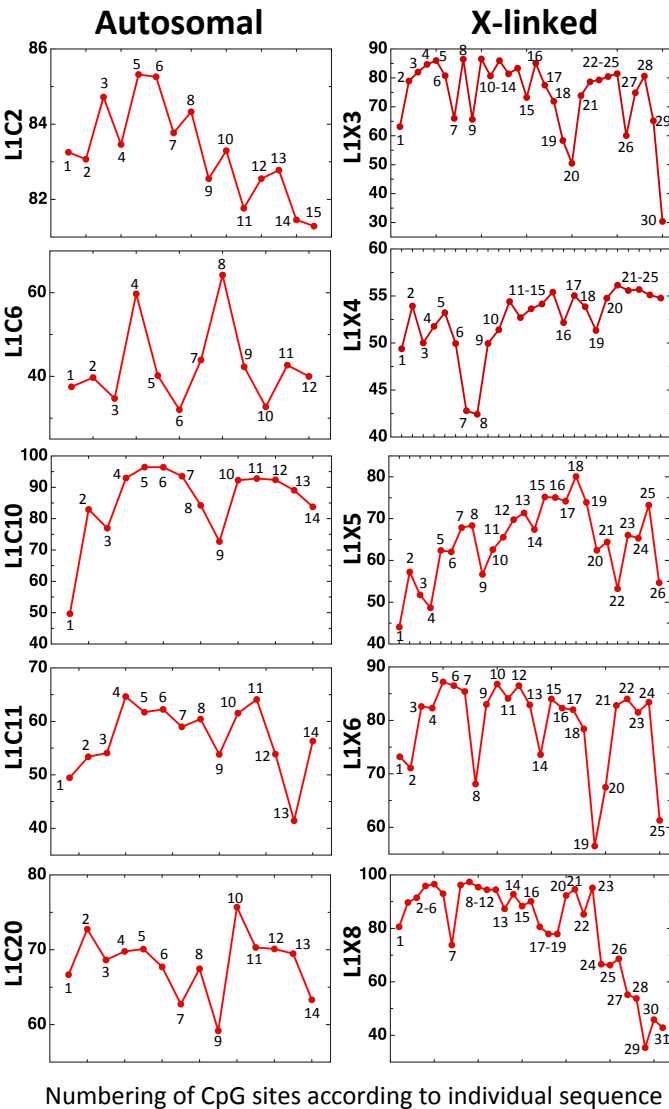

Supplement: Additional file 13: — Average of methylation levels of six NGS sequenced tumor samples at individual CpGs: (A) per patient and (B) per region. Autosomal and X-linked loci were analyzed separately. [file 13148_2015_51_MOESM13_ESM.pdf]

A) X-linked loci

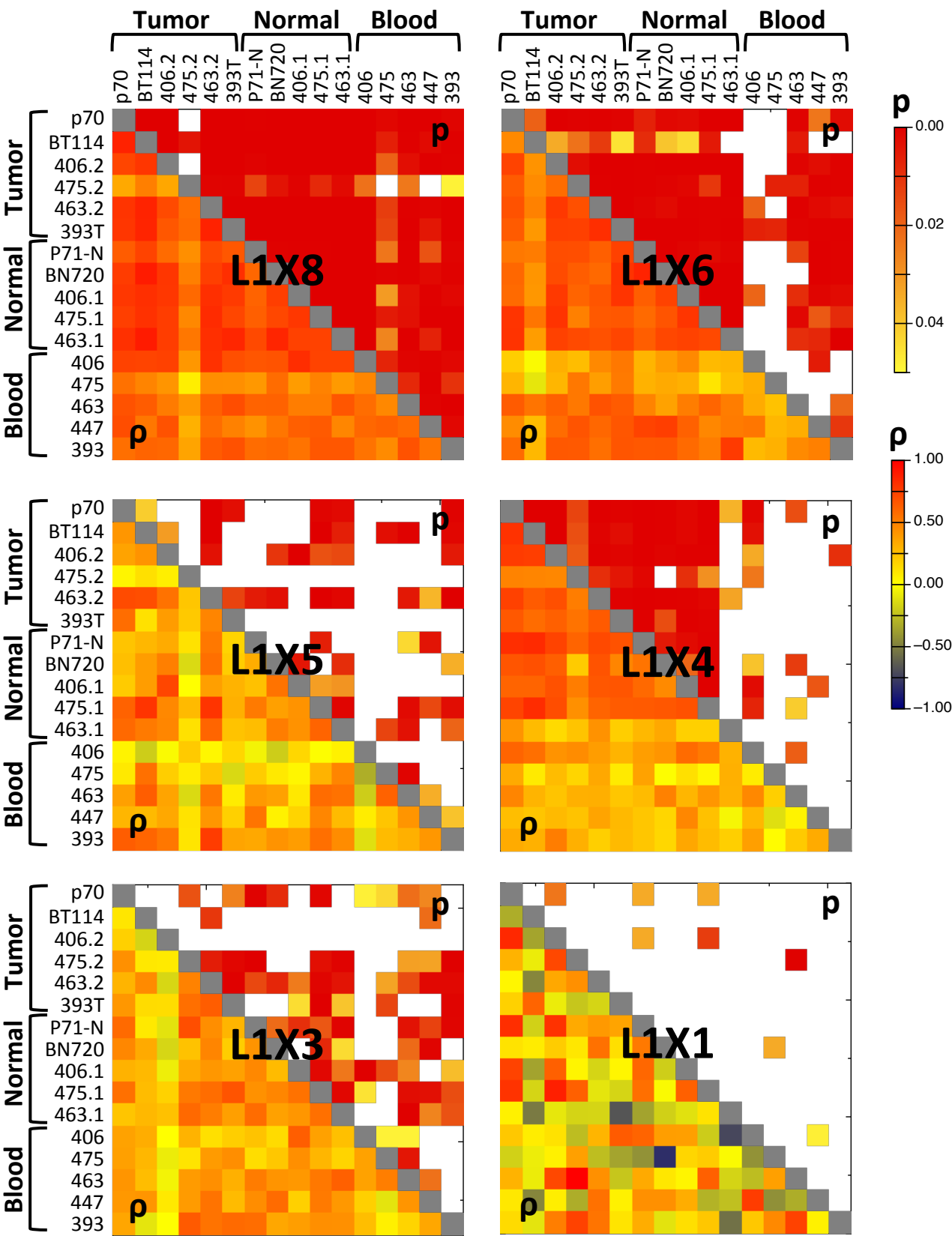

B) Autosomal

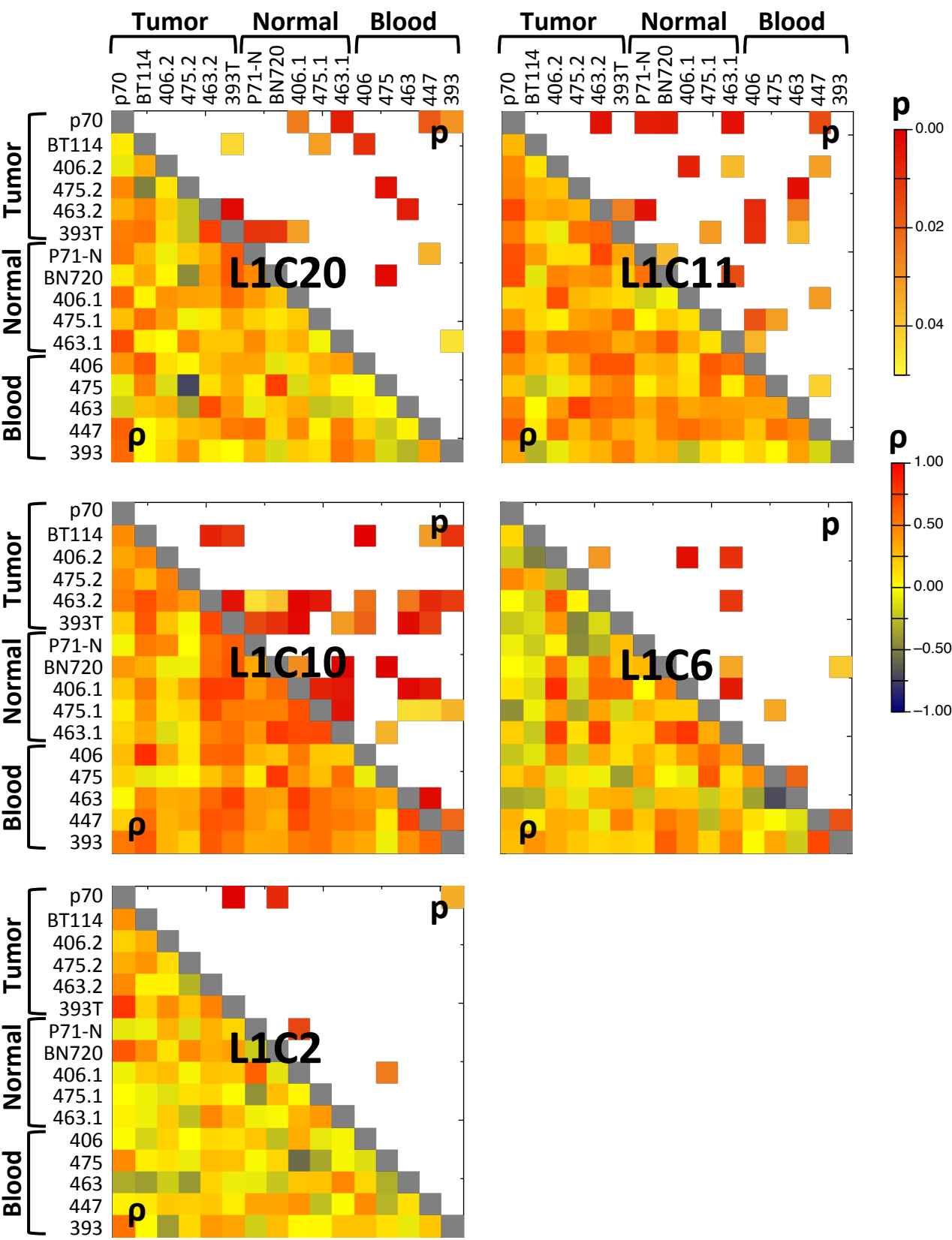

Supplement: Additional file 14: — Inter-sample correlation between average CpG methylation at a given L1 sequence: (A) X-linked loci and (B) autosomal loci. All samples studied by massive parallel sequencing were compared to each others. The rho Spearman correlation (lower left triangle) and the p values (upper right triangle) are represented by heat map boxes, one for each region. [file 13148_2015_51_MOESM14_ESM.pdf]

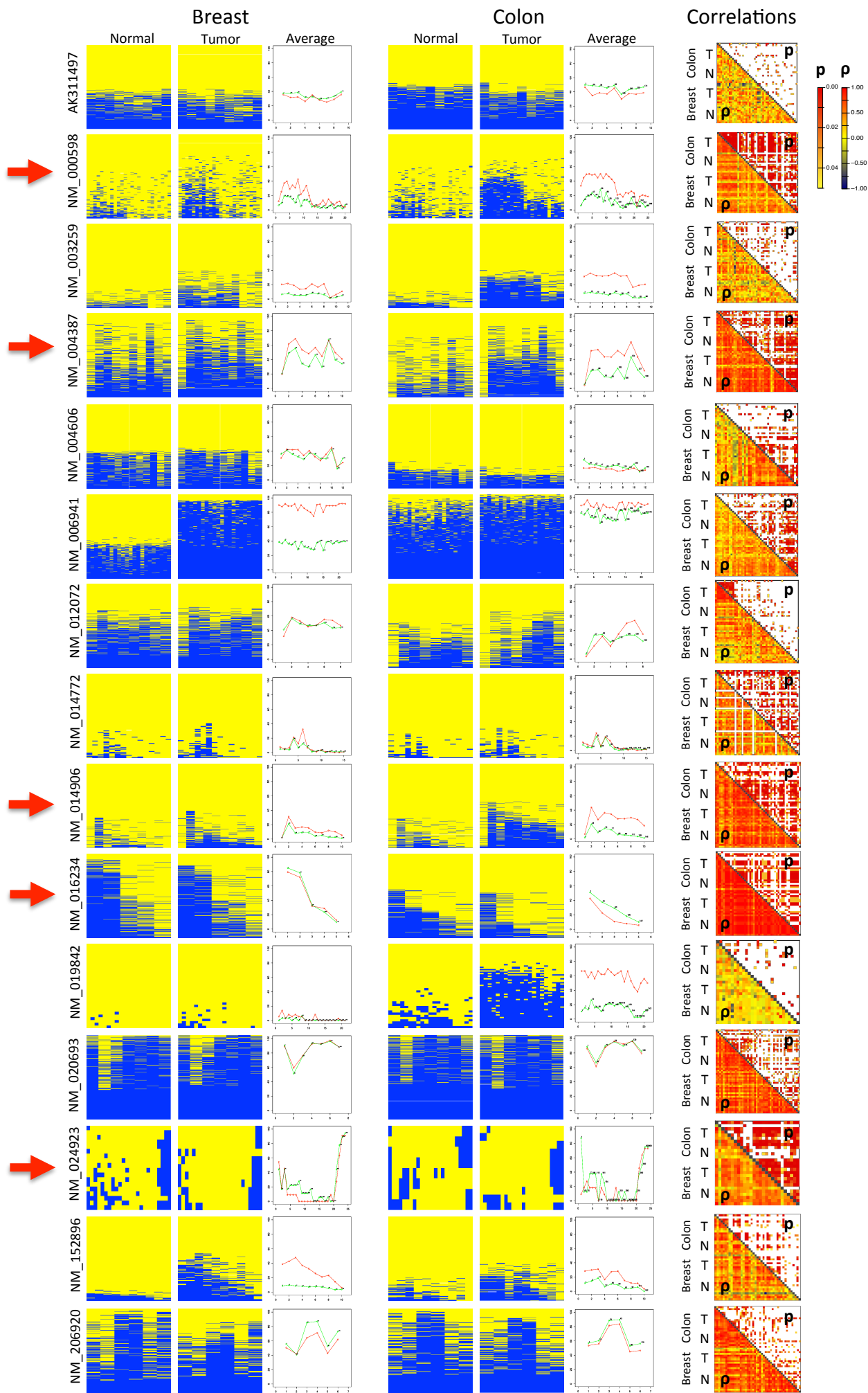

Supplement: Additional file 15: — Summary representation of massive parallel sequencing results of selected CpG islands in breast and colon cancer reported by Varley et al . [21]. The left, middle, and right part represent breast, colon, and correlation data, respectively. Heat maps representing combinations of all samples in one group are shown in yellow (unmethylated) and blue (methylated), whereby each column represents one CpG site and each line represent one sequence reads. The average of both the tumor and the healthy samples are shown to the right of the graph. Horizontal red arrows indicate the regions with the highest inter-sample correlations indicating a specific methylation pattern present across different samples, different tissues, and different disease status. [file 13148_2015_51_MOESM15_ESM.pdf]

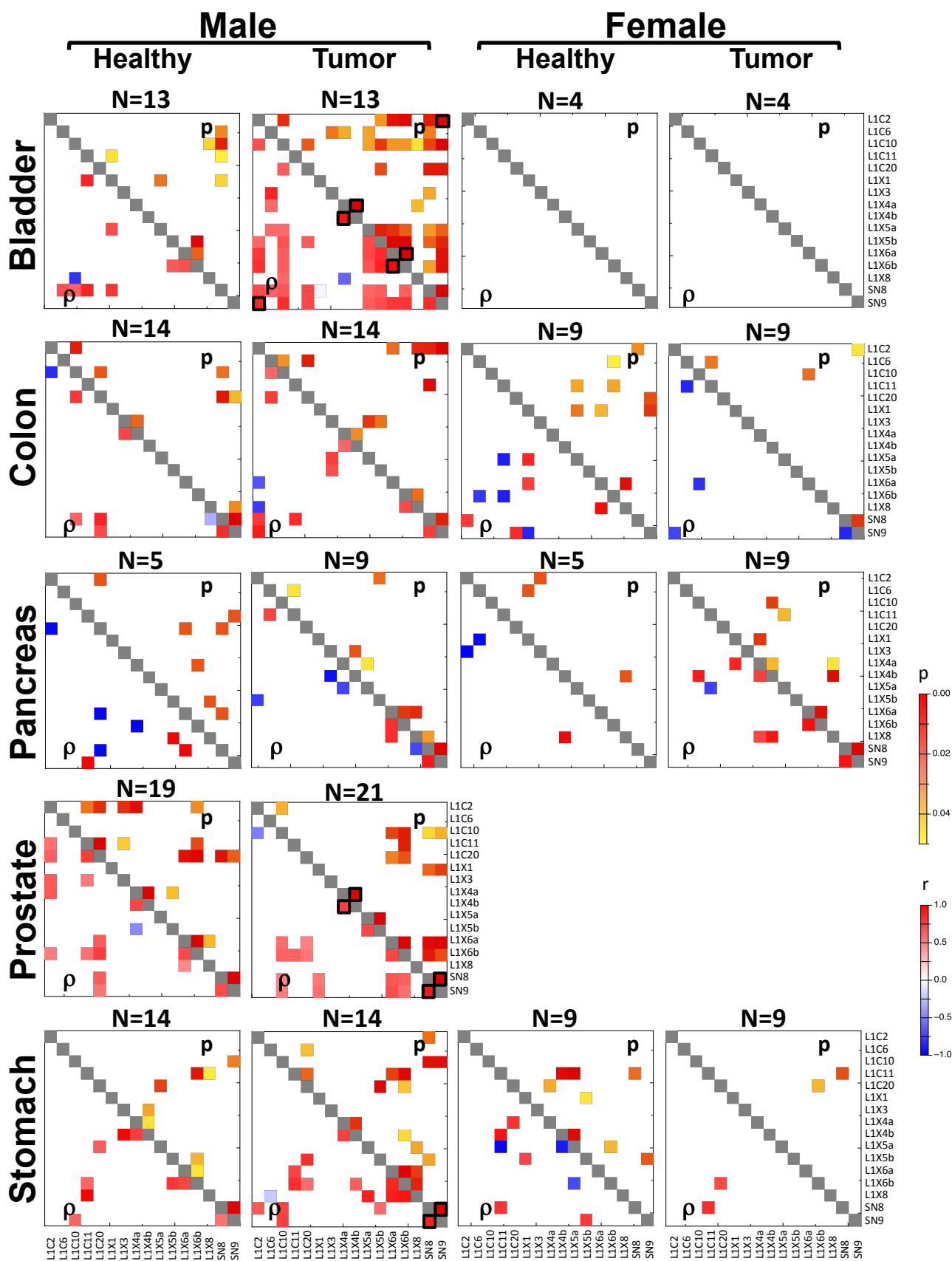

Supplement: Additional file 16: — Inter-locus correlations of all LINE-1 specific loci (based on pyrosequencing data) and the global LINE-1 assay (SN8 and SN9 based on SIRPH data). Inter-locus correlation based on the average within one region was separately performed for healthy tissue samples and tumors and for male and female. In the right upper and the low left triangles, p values and Spearman rho values are represented in a heat map. Only significant p value correlations are depicted. The significant correlations after Bonferroni corrections for multiple testing are highlighted in black squares. The number of sample used in each group is given above the correlation boxes. [file 13148_2015_51_MOESM16_ESM.pdf]
