# Supplementary material for: Inter-locus as well as intra-locus heterogeneity in LINE-1 promoter methylation in common human cancers suggests selective demethylation pressure at specific CpGs
Source: Clin Epigenetics. 2015 Mar 1;7(1):17. doi: 10.1186/s13148-015-0051-y (PMC4367886; doi:10.1186/s13148-015-0051-y)

Bladder (paired samples)

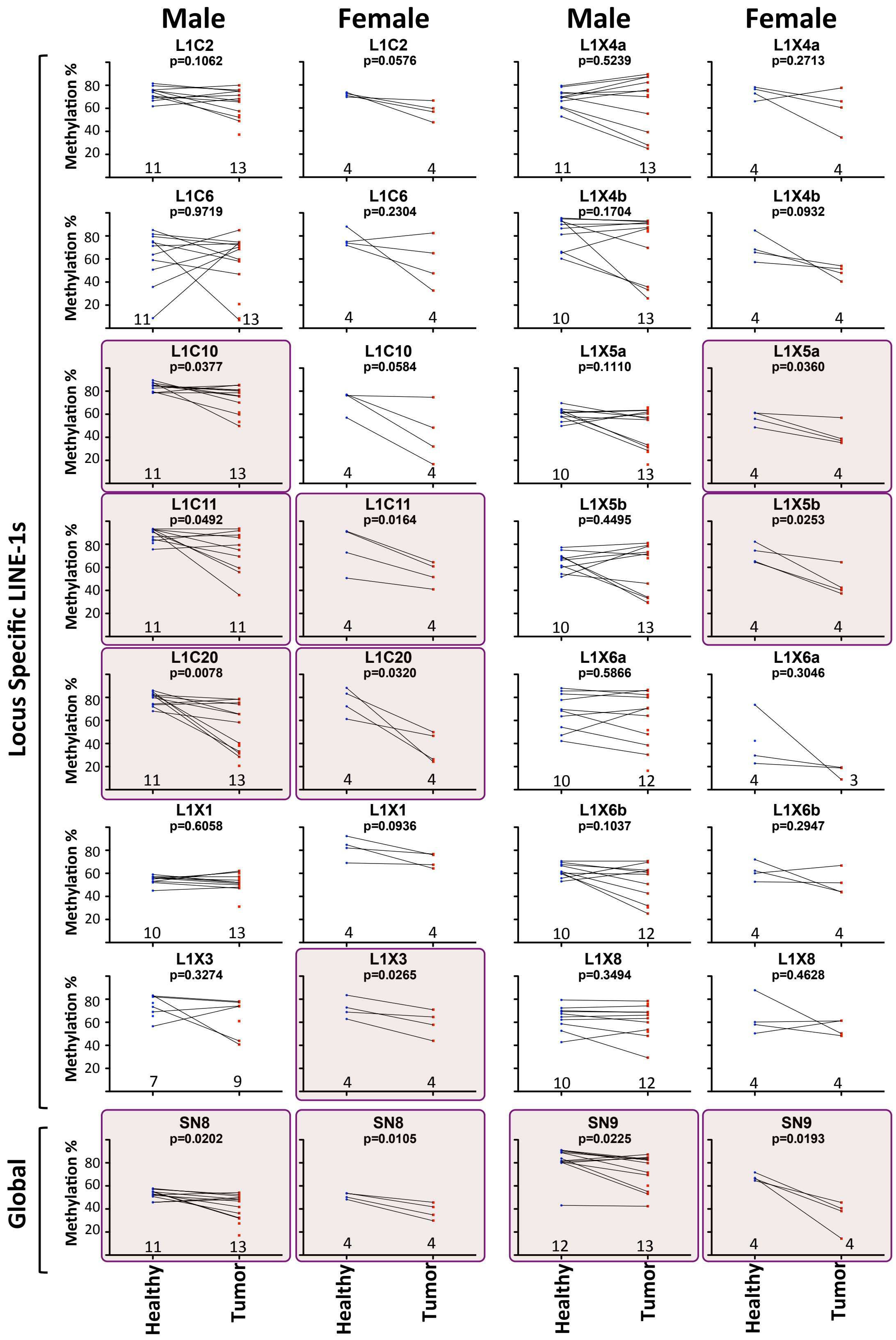

Stomach (paired samples)

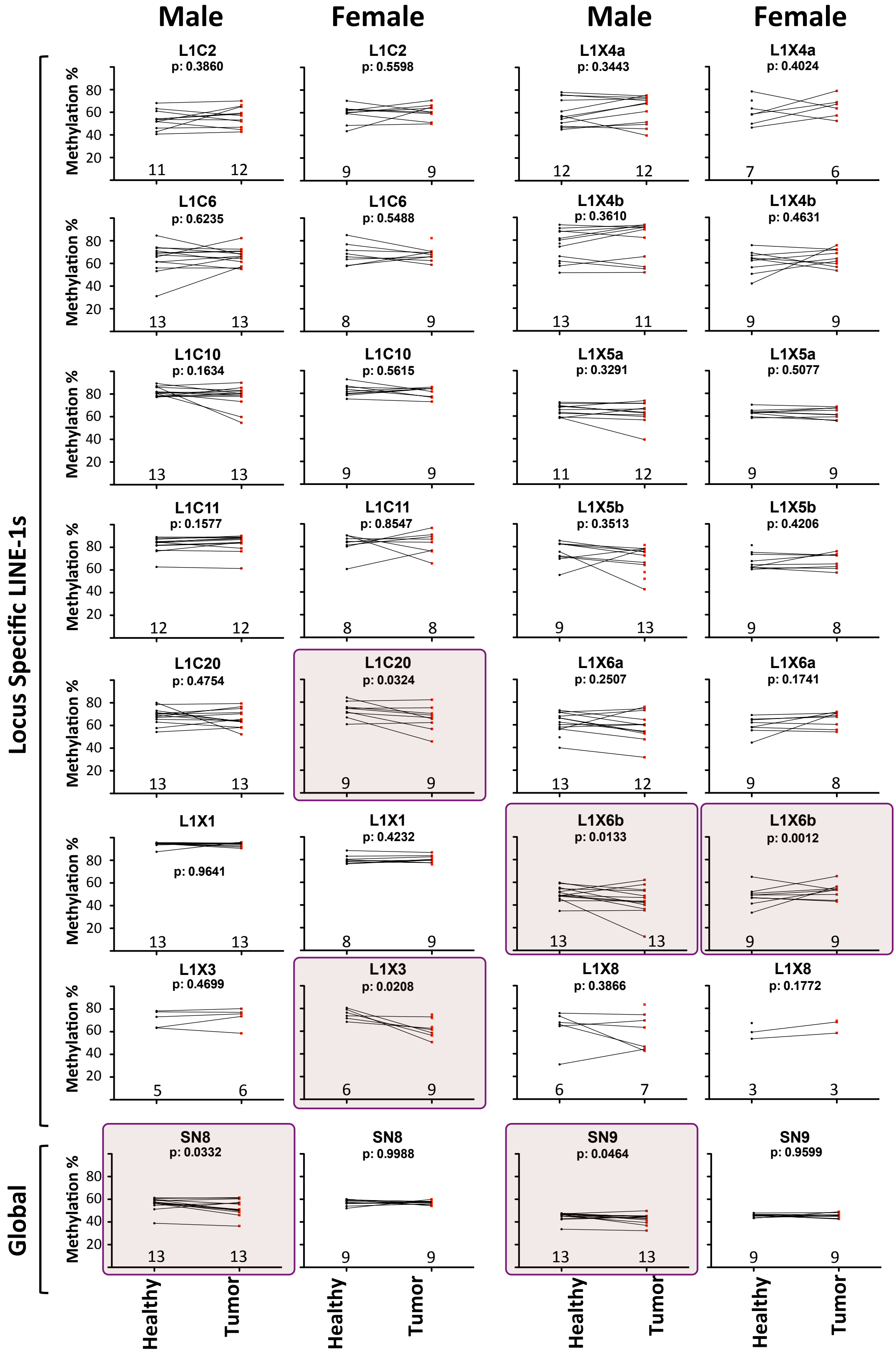

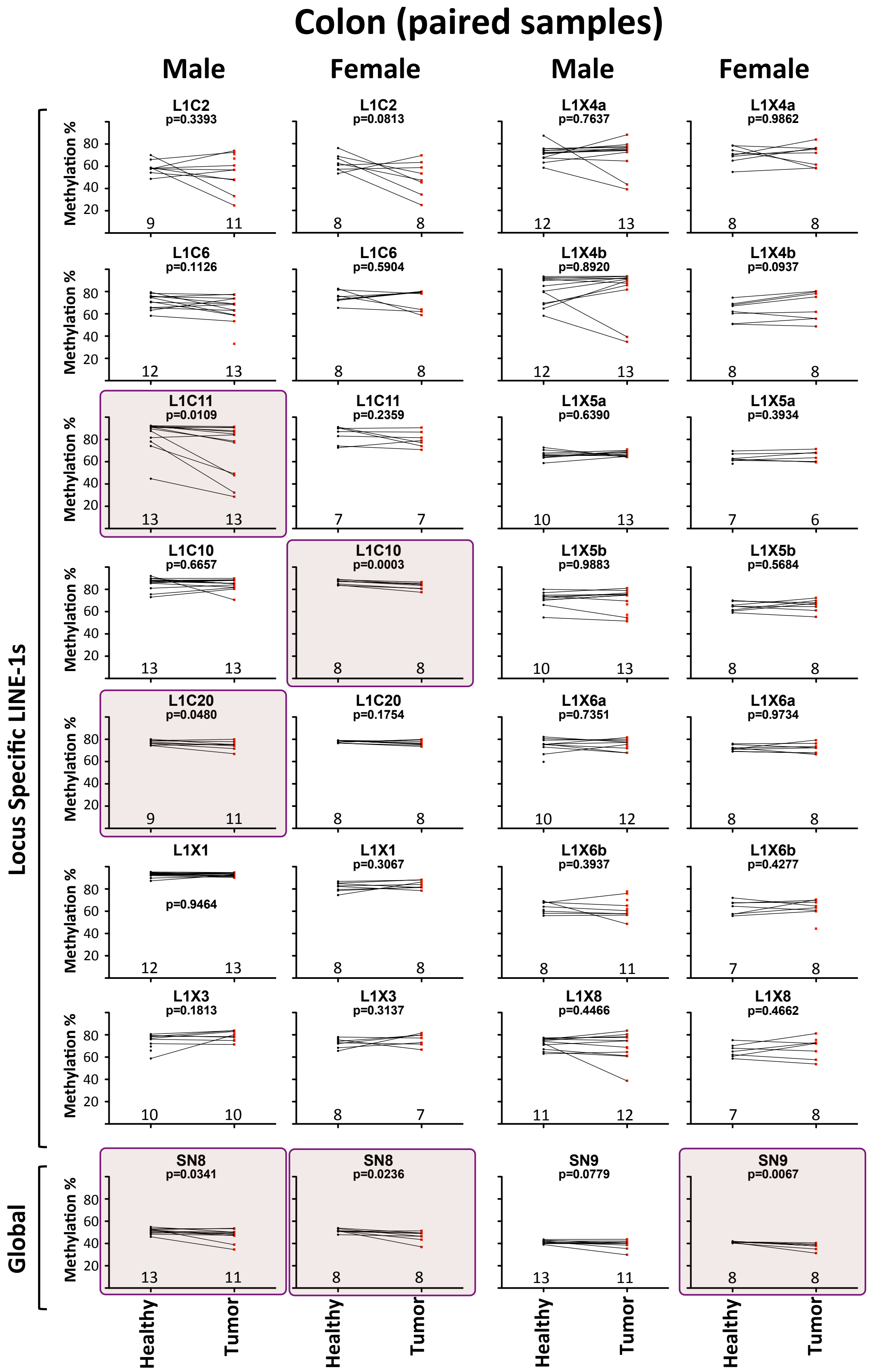
Colon (paired samples)
Male
Female
Male
Female
Locus Specific LINE-1s
Global
Methylation %
L1C2 p=0.3393
L1C2 p=0.0813
L1X4a p=0.7637
L1X4a p=0.9862
L1C6 p=0.1126
L1C6 p=0.5904
L1X4b p=0.8920
L1X4b p=0.0937
L1C11 p=0.0109
L1C11 p=0.2359
L1X5a p=0.6390
L1X5a p=0.3934
L1C10 p=0.6657
L1C10 p=0.0003
L1X5b p=0.9883
L1X5b p=0.5684
L1C20 p=0.0480
L1C20 p=0.1754
L1X6a p=0.7351
L1X6a p=0.9734
L1X1 p=0.9464
L1X1 p=0.3067
L1X6b p=0.3937
L1X6b p=0.4277
L1X3 p=0.1813
L1X3 p=0.3137
L1X8 p=0.4466
L1X8 p=0.4662
SN8 p=0.0341
SN8 p=0.0236
SN9 p=0.0779
SN9 p=0.0067
Healthy
Tumor

Prostate (paired samples)

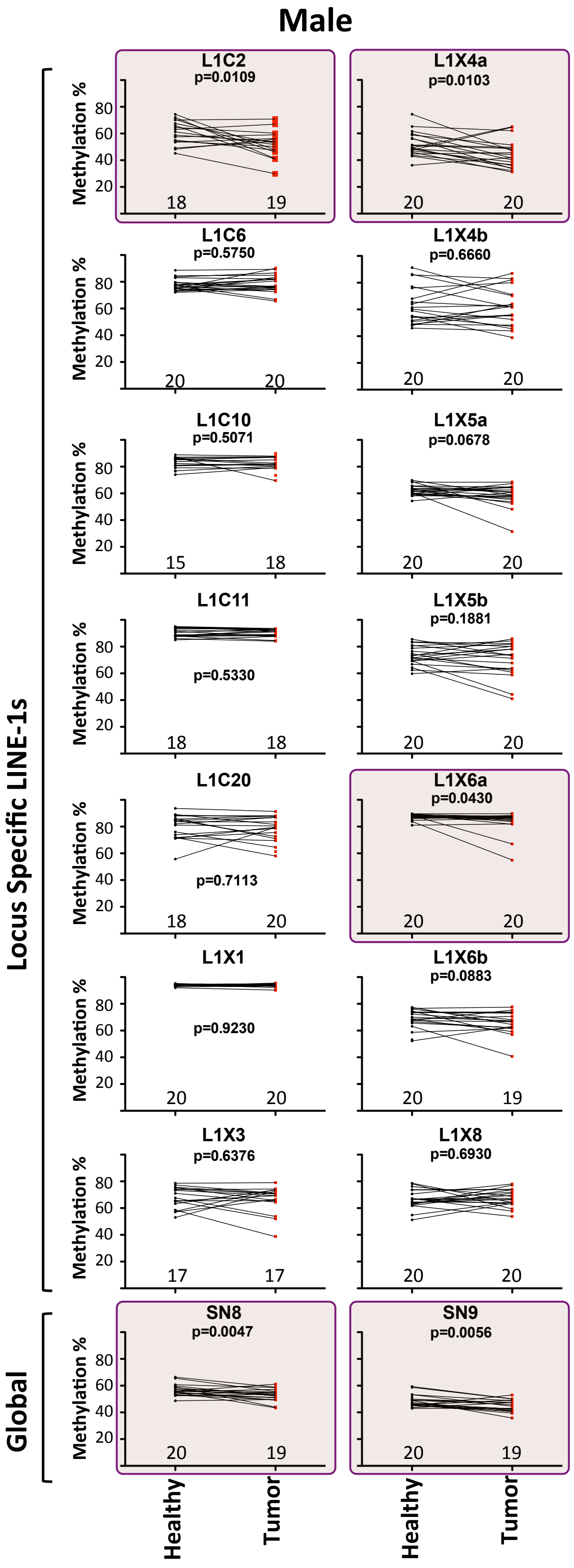

Pancreas (non paired samples)

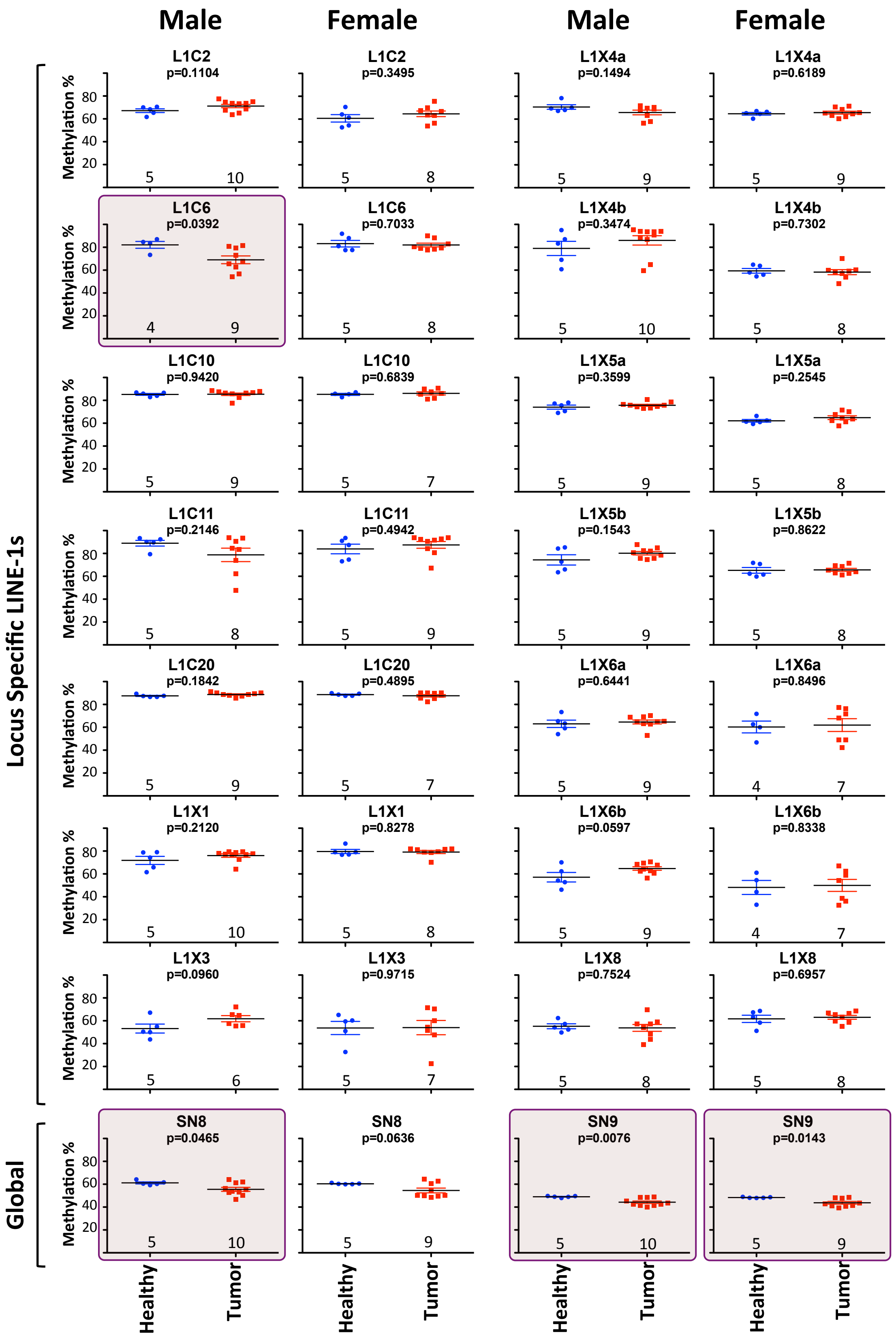

Supplement: Additional file 4: — Pair-wise comparisons of paired tissue (normal vs. cancer) samples of (A) bladder, (B) stomach, (C) colon, (D) prostate, and (E) non-paired sample of pancreatic cancer. Both methylation data from locus-specific LINE-1 (14 loci) and the global LINE-1 (two) assays are shown; male and female data are analyzed separately. Mann-Whitney test p values are indicated under the name of the locus; a transparent pink box highlights loci showing statistical significance. However, after correction for multiple testing (significance threshold of p < 0.00313) only L1X6b in stomach and L1C10 in colon remained significant. The number of samples analyzed for each healthy or tumor group at each loci is given below the scatter plot. [file 13148_2015_51_MOESM4_ESM.pdf]
