# Supplementary material for: Inter-locus as well as intra-locus heterogeneity in LINE-1 promoter methylation in common human cancers suggests selective demethylation pressure at specific CpGs
Source: Clin Epigenetics. 2015 Mar 1;7(1):17. doi: 10.1186/s13148-015-0051-y (PMC4367886; doi:10.1186/s13148-015-0051-y)
Supplement: Additional file 5: — Simple statistics on distribution of affected loci (A) Distribution and number of hypo/hyper-methylated LINE-1 loci in each patient (sorted in increasing numbers of affected loci). (B) Histograms showing the number of tumor tissues affected at a given number of loci. Hyper and hypo- methylated are defined as methylation differences between paired tissues of >10% and <10%, respectively. [file 13148_2015_51_MOESM5_ESM.pdf]

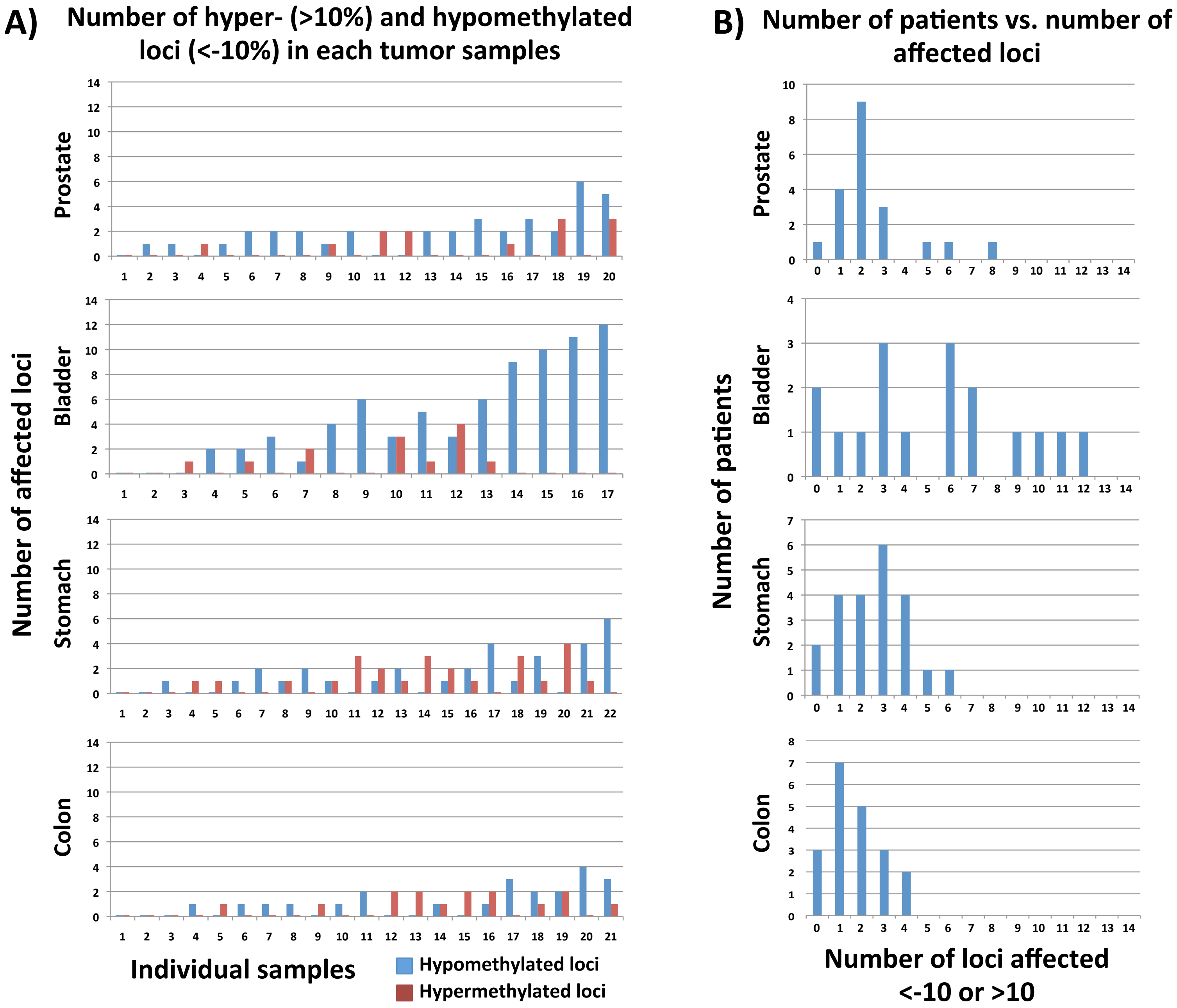

A) Number of hyper- (>10%) and hypomethylated loci (<-10%) in each tumor samples
Number of affected loci
Prostate
Bladder
Stomach
Colon
Individual samples
Hypomethylated loci
Hypermethylated loci
B) Number of patients vs. number of affected loci
Number of patients
Prostate
Bladder
Stomach
Colon
Number of loci affected <-10 or >10
